# Targeting the Sonic Hedgehog Pathway to Suppress the Expression of the Cancer Stem Cell (CSC)—Related Transcription Factors and CSC-Driven Thyroid Tumor Growth

**DOI:** 10.3390/cancers13030418

**Published:** 2021-01-22

**Authors:** Yurong Lu, Yiwen Zhu, Shihan Deng, Yuhuang Chen, Wei Li, Jing Sun, Xiulong Xu

**Affiliations:** 1 Institute of Comparative Medicine, College of Veterinary Medicine, Yangzhou University, Yangzhou 225009, Jiangsu, China; lyr1308065453@163.com (Y.L.); yiwen.zhu@sjtu.edu.cn (Y.Z.); qingxue1998@163.com (S.D.); chenghcn@163.com (Y.C.); sunj@yzu.edu.cn (J.S.); 2 College of Medicine, Yangzhou University, Yangzhou 225009, Jiangsu, China; weili@yzu.edu.cn; 3 Jiangsu Co-innovation Center for Prevention and Control of Important Animal Infectious Diseases and Zoonosis, Yangzhou University, Yangzhou 225009, Jiangsu, China

**Keywords:** thyroid cancer, BMI1, SOX2, sonic hedgehog, Gli1, Snail

## Abstract

**Simple Summary:**

Poorly differentiated and anaplastic thyroid cancers respond poorly to surgery, radiation, and hormone therapy. Cancer stem cells play an important role in tumor growth, drug resistance, and recurrence. This study focuses on how the sonic hedgehog (Shh) pathway maintains thyroid cancer stem cell self-renewal and whether it can be targeted for anticancer therapy. The authors report that the Shh pathway regulates the expression of BMI1 and SOX2, two genes involved in stem cell self-renewal, and that targeting the Shh pathway has little effect on thyroid tumor xenografts but can inhibit the growth of tumor xenografts derived from thyroid cancer stem cells. This study advances the knowledge on how thyroid cancer stem cells regenerate and highlights the potential therapeutic values of targeting the Shh pathway.

**Abstract:**

The sonic hedgehog (Shh) pathway plays important roles in tumorigenesis, tumor growth, drug resistance, and metastasis. We and others have reported earlier that this pathway is highly activated in thyroid cancer. However, its role in thyroid cancer stem cell (CSC) self-renewal and tumor development remains incompletely understood. B lymphoma Mo-MLV insertion region 1 homolog (BMI1) and SRY-Box Transcription Factor 2 (SOX2) are two CSC-related transcription factors that have been implicated in promoting CSC self-renewal. The objective of our current investigation was to determine the role of the Shh pathway in regulating *BMI1* and *SOX2* expression in thyroid cancer and promoting thyroid tumor growth and development. Here we report that inhibition of the Shh pathway by Gli1 siRNA or by cyclopamine and GANT61 reduced BMI1 and SOX2 expression in SW1736 and KAT-18 cells, two anaplastic thyroid cancer cell lines. The opposite results were obtained in cells overexpressing Gli1 or its downstream transcription factor Snail. The Shh pathway regulated *SOX2* and *BMI1* expression at a transcriptional and post-transcriptional level, respectively. GANT61 treatment suppressed the growth of SW1736 CSC-derived tumor xenografts but did not significantly inhibit the growth of tumors grown from bulk tumor cells. Clinicopathological analyses of thyroid tumor specimens by immunohistochemical (IHC) staining revealed that BMI1 and SOX2 were highly expressed in thyroid cancer and correlated with Gli1 expression. Our study provides evidence that activation of the Shh pathway leads to increased BMI1 and SOX2 expression in thyroid cancer and promotes thyroid CSC-driven tumor initiation. Targeting the Shh pathway may have therapeutic value for treating thyroid cancer and preventing recurrence.

## 1. Introduction

Thyroid cancer is the most common endocrine malignancy [1,2]. Thyroid cancer can be divided into several different pathological types including well-differentiated papillary (PTCs), follicular (FTCs), medullary (MTCs), Hürthle cell carcinomas (HTCs), and poorly differentiated or anaplastic thyroid carcinomas (ATCs) [2]. PTCs account for more than 80% of all thyroid cancers [2]. Surgery, thyroid hormone therapy, and radioiodine can cure most differentiated thyroid cancers (PTC and FTC), but are not effective for poorly differentiated thyroid cancer [3]. Approximately 15–20% of thyroid cancer patients develop recurrence in their lifetime [2]. Currently, there is no effective therapy for treating ATC, which is almost always fatal, with a mean survival of only 2–6 months [4]. 

A small fraction of aldehyde dehydrogenase (ALDH)-positive cells, which constitute approximately 1–3% of total thyroid tumor cells, represents cancer stem cells (CSCs) [5]. Poorly differentiated or undifferentiated thyroid cancer contains a relatively higher percentage of ALDH-positive CSCs than benign adenomas and well-differentiated thyroid cancers [5]. The stage-specific embryonic antigen-1 (SSEA-1) has been identified as an alternative marker for thyroid CSCs [6]. SSEA-1-postive thyroid CSCs express high levels of stem cell-related genes such as Nanog, SOX2, and Oct4, and are resistant to 5-fluorouracil cytotoxicity [6]. Thyroid CSCs are highly tumorigenic. Injection of several thousand ALDH-positive cells into immunodeficient mice leads to tumor implantation and growth [5]. Although there has been growing interest in targeting CSCs as a novel therapeutic strategy to control cancer metastasis and recurrence, whether targeting thyroid CSCs impacts thyroid tumor development is not known. 

The Shh pathway plays an important role in CSC self-renewal, tumor cell growth, drug resistance, metastasis, and recurrence [7]. The Shh pathway involves the binding of a hedgehog ligand (Sonic, Indian, or Desert) to their shared Patched (Ptch) receptor, a twelve-pass transmembrane protein. Ligand binding to the Ptch receptor triggers the translocation of Smoothened (Smo), a G-protein-coupled seven-pass transmembrane protein, into the primary cilia, where it becomes active [8,9,10]. Smo activation leads to the disruption of Sufu with Gli1, a latent zinc-finger transcription factor [11,12]. Gli1 is then translocated to the nucleus where it induces the expression of many target genes such as Snail, a transcription factor that plays a pivotal role in epithelial-to-mesenchymal transition (EMT) and metastasis. The Shh pathway regulates the self-renewal of CSCs [13] in breast cancer [14,15], embryonal rhabdomyosarcoma CSCs [16], multiple myeloma [17], leukemia [18,19], and glioblastoma [20,21]. Our prior studies have shown that the Shh pathway promotes thyroid CSC self-renewal [22], renders thyroid tumor cells more resistant to killing by radiation [22], and increases the metastatic potential of thyroid cancer cells [23]. How the Shh pathway maintains the self-renewal of thyroid CSCs remains unknown. 

BMI1, a transcription factor of the polycomb gene group, is overexpressed in a variety of malignancies [24]. BMI1 regulates gene expression by chromatin modification and plays an important role in tumorigenesis [24]. BMI1 downregulates PTEN expression in nasopharyngeal cancers [25] and increases the proliferation of hepatocellular carcinomas [26,27]. BMI1 stimulates CSC self-renewal in hepatocellular carcinoma [26,27], pancreatic cancer [28,29], and head and neck squamous cell carcinoma [30,31]. BMI1 induces an invasive signature that promotes metastasis and chemoresistance in melanoma [32]. Whether BMI1 expression is dysregulated in thyroid cancer and has a role in thyroid CSC self-renewal remains to be investigated.

SOX2 is another transcription factor that contributes to normal and cancer stem cell self-renewal [33]. SOX2 is overexpressed in malignant glioblastoma [34] and in lung and basal cell-like breast cancer [35,36]. SOX2 is required for tumor cells to retain the characteristics of CSCs such as being highly tumorigenic and metastatic [37]. Castration-resistant BMI1^+^SOX2^+^ prostate cancer cells play an important role in prostate cancer recurrence [38]. SOX2 expression is elevated in ATCs and SW1736 cells and plays a crucial role in chemosensitivity to cisplatin and doxorubicin [39]. Whether SOX2 expression is increased in other types of thyroid cancer has not been investigated. Our present study investigates the expression and regulation of BMI1 and SOX2 by the Shh pathway. Here we report that BMI1 and SOX2 were highly expressed in thyroid cancer and correlated with Gli1 levels. We further show that the Shh pathway regulated BMI1 and SOX2 expression at a post-transcriptional and transcriptional level, respectively. Thyroid tumor growth was retarded in GANT61-treated mice. Our study provides evidence that the Shh pathway regulates BMI1 and SOX2 expression and could be potentially targeted for anti-thyroid cancer therapy. 

## 2. Results

### 2.1. GANT61 and Cyclopamine Inhibit BMI1 and SOX2 Expression 

**Figure 1:**
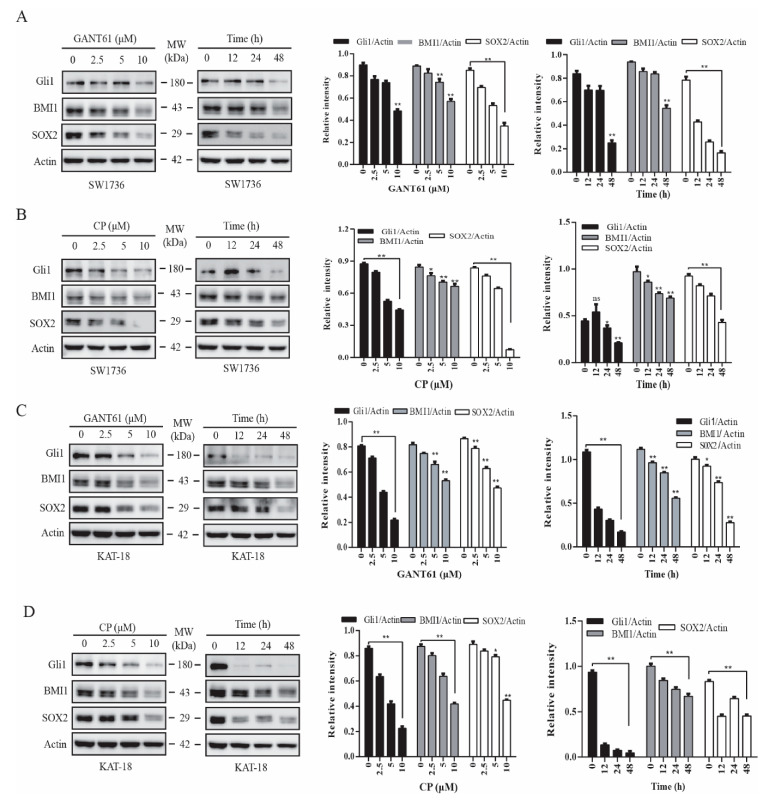
Inhibition of the sonic hedgehog (Shh) pathway suppressed BMI1 and SOX2 expression. SW1736 (**A**,**B**) and KAT-18 (**C**,**D**) cells were incubated for 48 h in the absence or presence of the indicated concentrations of cyclopamine or GANT61 or incubated with GANT61 (10 µM) (**A**,**C**) or cyclopamine (CP) (10 µM) (**B**,**D**) for the indicated lengths of time. Cell lysates were prepared and analyzed for Gli1, BMI1, SOX2, and actin expression by Western blot. Relative protein levels were analyzed by quantification of the density of the protein bands with NIH Image-J software and presented as bar graphs. Data are mean ± standard deviation (SD) of three experiments. * p < 0.05, ** p < 0.01, compared to untreated control. ns: not significant.

BMI1 and SOX2 are two transcription factors implicated in regulating stem cell self-renewal [24,33]. We reported earlier that inhibition of the Shh pathway leads to the suppression of thyroid CSC self-renewal [22]. Here we tested if the inhibitors of the Shh pathway also downregulated the expression of BMI1 and SOX2. GANT61 (Figure 1A,C) and cyclopamine (Figure 1B,D), an inhibitor of Gli1 and Smo, respectively, inhibited BMI1 and SOX2 expression in SW1736 (Figure 1A,B) and KAT-18 (Figure 1C,D) cells in a dose- and time-dependent manner. Both GANT61 and cyclopamine inhibited SOX2 expression more than BMI1 in both SW1736 and KAT-18 cells. Both inhibitors suppressed the expression of Gli, BMI1, and SOX2 proteins in KAT-18 cells slightly better than in SW1736 cells. GANT61 also inhibited BMI1 and SOX2 expression in WRO82 cells, a third thyroid cancer cell line with wild-type BRAF gene (Figure S1). Of note, Gli1 upregulates the expression of Gli1 itself and several other molecules in the Shh pathway in an autocrine manner [40], which explains why the inhibitors of the Shh pathway also downregulated Gli1 levels (Figure 1).

### 2.2. The Shh Pathway and Snail Regulates BMI1 and SOX2 Expression

**Figure 2:**
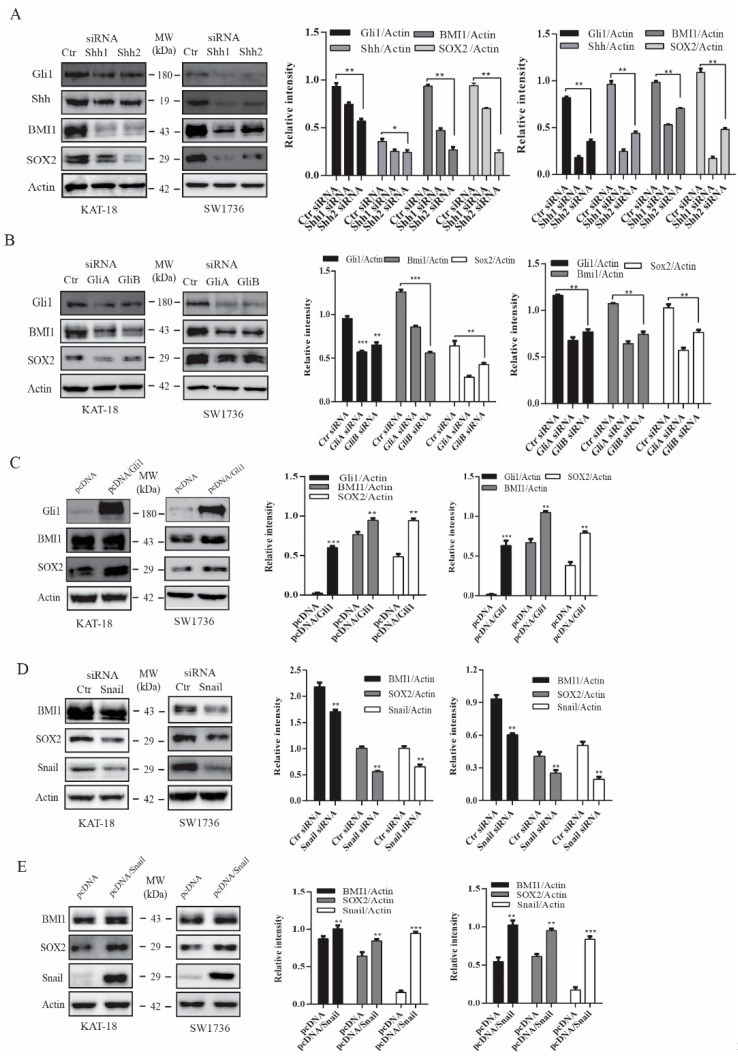
The Shh pathway regulated BMI1 and SOX2 expression. KAT-18 and SW1736 cells transfected with Shh (**A**), Gli1 (**B**), or Snail (**D**) siRNA or transfected with an empty vector or the vector encoding Gli1 (**C**) or Snail (**E**) were analyzed for Shh, Gli1, BMI1, and SOX2 expression by Western blot. Scramble siRNA was used as a negative control. Actin was included as a loading control. Relative protein levels were analyzed by quantification of the density of the protein bands with NIH Image-J software and presented as bar graphs. Data are mean ± SD of three experiments. * p < 0.05, ** p < 0.01, *** *p* < 0.001 compared to the control.

Next, we tested if silencing Shh and Gil1 also led to the downregulation of BMI1 and SOX2 expression. Shh and Gli1 siRNA, two sets for each gene, effectively suppressed the expression of their corresponding genes in both KAT and SW1736 cells (Figure 2A,B). Shh siRNA also downregulated Gli1 expression due to its autocrine regulation (Figure 2A). Gli1 and Shh siRNA significantly suppressed BMI1 and SOX2 expression in these two cell lines (Figure 2A,B). We then determined if Gli1 overexpression increased SOX2 and BMI1 expression. As shown in Figure 2C, Gli1 was overexpressed in SW1736 and KAT-18 cells transfected with a human Gli1 expression vector. Gli1 overexpression led to increased BMI1 and SOX2 expression (Figure 2C). Snail is a transcription factor directly regulated by Gli1 [41]. Our prior study has shown that inhibition of the Shh pathway by GANT61 and cyclopamine or Gli1 silencing downregulates Snail expression [23]. Here we tested if Snail played a role in regulating BMI1 and SOX2 expression. As shown in Figure 2D, Snail expression was downregulated in KAT-18 and SW1736 cells transfected with Snail siRNA. Snail siRNA suppressed BMI1 and SOX2 in both cell lines. In contrast, Snail overexpression significantly increased Snail, BMI1, and SOX2 expression in KAT-18 and SW1736 cells (Figure 2E). Of note, Snail had a relatively weak effect on BMI1 expression, particularly in KAT-18 cells. Nevertheless, these observations collectively suggested that the Shh pathway promotes SOX2 and BMI1 expression in thyroid tumor cell lines.

### 2.3. The Shh Pathway Regulates SOX2 and BMI1 by Different Mechanisms

**Figure 3:**
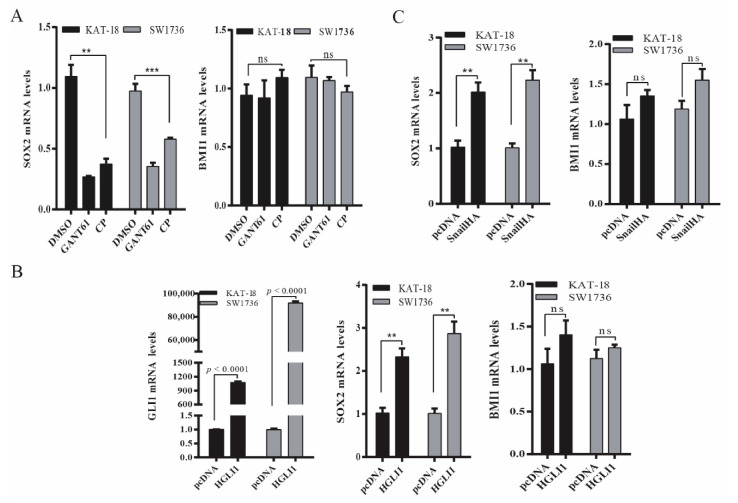
The Shh pathway regulated SOX2 and BMI1 mRNA expression. (**A**) KAT-18 and SW1736 cells were incubated for 48 h in the absence or presence of GANT61 (10 µM) or cyclopamine (10 µM) for 48 h. (**B**) KAT-18 and SW1736 cells were transfected with the empty expression vector or the vector encoding Gli1 (**B**) or Snail (**C**). After incubation for 48 h, total RNA was extracted and analyzed for SOX2, BMI1, and Gli1 mRNA levels by RT-PCR. GAPDH was included as an internal control. Data represent the results of mean ± SD of three independent experiments. ** *p* < 0.01; *** *p* < 0.001, ns: not significant when compared to the control.

Next, we determined if the Shh pathway regulated BMI1 and SOX2 expression at a transcriptional level. As shown in Figure 3A, SOX2 mRNA levels were significantly decreased in SW1736 and KAT-18 cells treated with GANT61 (10 µM) or cyclopamine (10 µM) for 48 h. In contrast, BMI1 mRNA levels were not significantly changed in these two cell lines treated with GANT61 or cyclopamine (Figure 3A). Consistent with this observation, Gli1 (Figure 3B) and Snail (Figure 3C) overexpression significantly increased SOX2 mRNA levels but did not significantly change BMI1 mRNA levels. 

**Figure 4:**
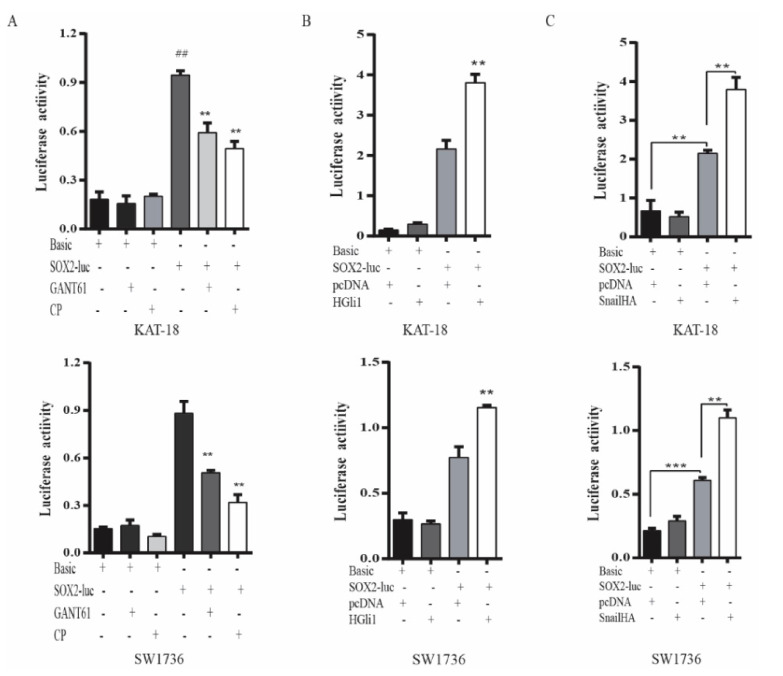
The Shh pathway regulated SOX2 promoter activity. (**A**) KAT-18 and SW1736 cells were transiently transfected with the pGL3/Basic or the SOX2 promoter-driven luciferase reporter (pGL3/SOX2-Luc) and incubated for 48 h in the absence or presence of GANT61 (10 µM) or cyclopamine (10 µM) for 48 h. (**B**) KAT-18 and SW1736 cells were transient transfected with the pGL3/Basic or the SOX2 promoter-driven luciferase reporter (pGL3/SOX2-Luc) plus the empty pcDNA3.1 vector or the expression vector encoding human Gli1 (**B**) or Snail (**C**) and incubated for 48 h. The cell lysates were prepared and analyzed for luciferase activity. Data represent the results of one of two experiments with similar results. ** *p* < 0.01, *** *p* < 0.001, compared to the untreated control; ^##^ *p*<0.01, compared to the pGL3/Basic control.

To verify that the Shh pathway regulated SOX2 expression at a transcriptional level, we investigated if the Shh pathway also regulated SOX2 promoter activity. As shown in Figure 4A, luciferase activity in KAT-18 and SW1736 cells transfected with the SOX2 promoter-driven reporter gene was significantly higher than in those transfected with the control pGL3/Basic vector. GANT61 and cyclopamine significantly decreased SOX2 promoter-driven luciferase activity in KAT-18 and SW1736 cells but had no effect on luciferase activity in those transfected with the pGL3/Basic vector (Figure 4A). Luciferase activity was also significantly higher in SW1736 cells co-transfected with the Gli1 or Snail expression vector than with the empty vector (Figure 4B,C). Gli1 co-transfection had no significant effect on luciferase activity in these two cell lines transfected with the pGL3/Basic vector.

### 2.4. GANT61 Suppresses the Growth of Thyroid CSC-Derived Tumors

**Figure 5:**
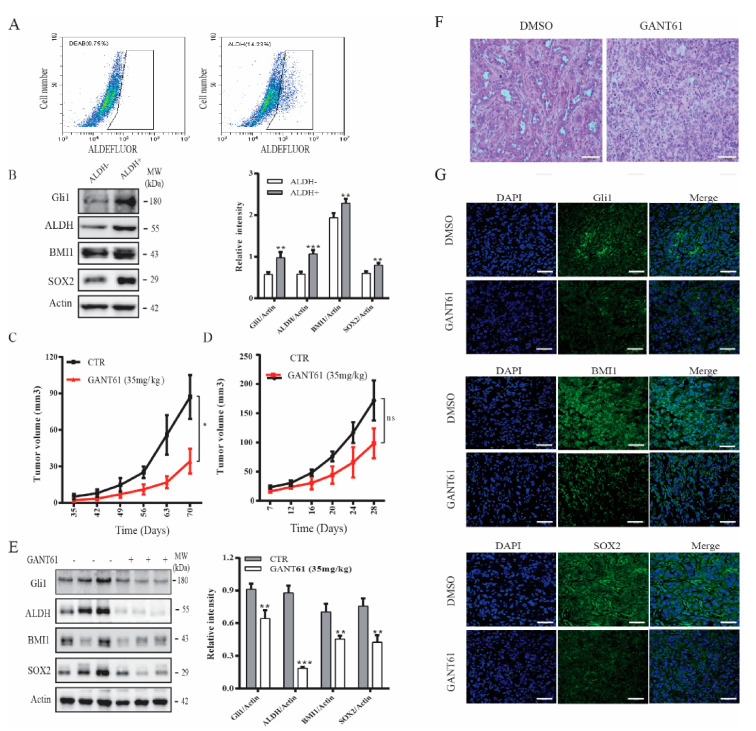
The Shh pathway promoted thyroid tumor development. (**A**) SW1736 cells were stained with ALDELUOR fluorescent dye and analyzed in flow cytometry. Diethylaminobenzaldehyde (DEAB), an inhibitor of ALDH, was added prior staining as a background control (left pane). ALDH-positive cells shown in the cropped area of the right panel and ALDH-negative cells (outside the cropped area) were collected separately. (**B**) Cell lysates prepared from ALDH-positive and ALDH-negative SW1736 cells sorted by flow cytometry in (**A**) were analyzed for BMI1, SOX2, Gli1 expression by Western blot. (**C**) ALDH-positive SW1736 cells (5 × 10^4^ cells/mouse, 8 mice per group) were injected subcutaneously into NCG mice. One week later, the mice were treated with the corn oil vehicle or GANT61 (35 mg/kg/day) daily for two weeks by intraperitoneal injection. Tumor volumes were measured twice weekly for 10 weeks. (**D**–**F**) Unsorted SW1736 cells (1 × 10^6^ cells/mouse, 8 mice per group) were injected subcutaneously into NCG mice. Three days later, mice were treated with the corn oil vehicle or GANT61 (35 mg/kg/day) daily for 3 weeks by intraperitoneal injection. Tumor volumes were calculated and statistically analyzed (**D**) Tumor tissues form mice were homogenized and analyzed for Gli1, BMI1, SOX2, and Actin expression by Western blot. The samples from three mice in the untreated or GANT61-treated groups are shown (**E**). Relative protein levels in the tumor tissues from all eight mice were analyzed by quantification of the density of the protein bands with NIH Image-J software and presented as bar graphs. * *p* < 0.05, ** *p* < 0.01, *** *p* < 0.001, ns: not significant. (**F**) The sections of paraffin-embedded tumor blocks from untreated or GANT61-treated mice were analyzed for histological appearance by H&E staining. Scale bar, 100 mm. (**G**) The frozen sections of tumor tissues from untreated or GANT61-treated mice were analyzed for BMI1, SOX2, and Gli1 expression by IF staining with their specific antibodies. Scale bar, 50 mm.

CSCs play an important role in tumor initiation [5]. Our prior study showed that the Shh pathway plays an important role in maintaining thyroid CSC self-renewal [22]. Here we tested if GANT61 could inhibit thyroid CSC-driven thyroid tumor development. We first analyzed if ALDH-positive CSCs enriched from SW1736 cells expressed higher levels of Gli1, SOX2, and BMI1. ALDH-positive and ALDH-negative cells from SW1736 cells were separately collected by flow cytometry. As shown in Figure 5A (right panel), SW1736 cells contained approximately 15% of ALDH-positive cells. However, ALDH-positive cells were not detectable in the presence of the ALDH-inhibitor diethylaminobenzaldehyde (DEAB) (Figure 5A, left panel). The levels of ALDH, BMI1, SOX2, and Gli1 were significantly higher in ALDH-positive than ALDH-negative cells (Figure 5B). The ALDH-positive SW1736 cells subcutaneously implanted in immunodeficient NCG (NOD/ShiLtJGpt-*Prkdc*^em26^Il2rg^em26^/Gpt) mice were more capable of developing xenograft tumors than the ALDH-negative cells (Table 1). Twenty percent of mice receiving 500 ALDH-positive cells developed tumors (Table 1). All five mice receiving 5 × 10^3^ ALDH-positive cells developed tumors. In contrast, at least 5 × 10^4^ ALDH-negative cells were needed to grow tumors in mice (Table 1). None of five mice receiving 5 × 10^3^ ALDH-negative cells developed tumors (Table 1).

Our prior study showed that cyclopamine and GANT61 possess weak antiproliferative activity on SW1736 cells [38,42]. Here we tested if GANT61 treatment differentially impacted the growth of thyroid tumors derived from ALDH-positive SW1736 cells and from unsorted bulk SW1736 cells. Since thyroid cancer comprises of a small fraction of cancer stem cells [5], we only evaluated the inhibitory effect of GANT61 on the growth of SW1736 tumor initiated from the unsorted cells. As shown in Figure 5C, tumor growth was effectively retarded in mice treated with GANT61 (35 mg/kg/day) for two weeks, starting one week after ALDH-positive cells were inoculated. In contrast, tumor growth was weakly but not significantly inhibited in mice receiving unsorted SW1736 cells, even though the mice were treated with GANT61 (35 mg/kg/d) during the entire experimental period (Figure 5D). Of note, the average size of the tumors derived from the nonsorted SW1736 cells was much larger than the average size of the tumors derived from the ALDH-positive SW1736 cells. This was because far fewer ALDH-positive cells (5 × 10^4^ cells/mouse) were injected subcutaneously than the nonsorted bulk SW1736 cells (1 × 10^6^ cells/mouse). Western blot analysis revealed that the levels of Gli1, BMI1, and SOX2 proteins in the tumor tissues from GANT61-treated mice were significantly lower than that from untreated controls (Figure 5E). H&E staining revealed that tumor cells in GANT61-treated mice appeared to be smaller with less cytoplasm (Figure 5F) than those in control mice. Immunofluorescence staining revealed that GANT61 treatment also decreased the expression of these three proteins in tumor tissues (Figure 5G). Normal mouse and rabbit IgG were used as negative controls. No specific signals were present (Figure S2).

### 2.5. Gli1 Expression Correlates with the Levels of SOX2 and BMI1 in Thyroid Cancer

**Figure 6:**
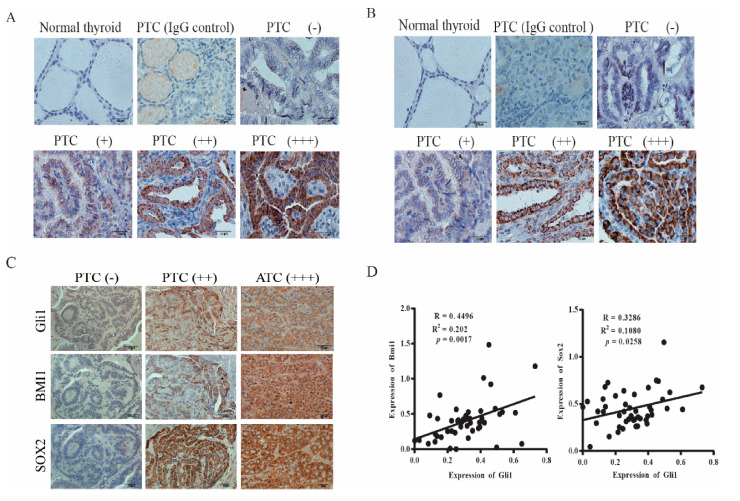
SOX2 and BMI1 expression in thyroid cancer. The sections of paraffin-embedded tissue blocks were analyzed for BMI1 (**A**) and SOX2 (**B**) expression by immunohistochemical (IHC) staining with their specific antibodies. A representative of normal thyroid follicles and papillary thyroid carcinoma (PTC) with different expression levels is given. Normal rabbit IgG was included as a negative control. BMI1 and SOX2 expression in a representative PTC graded as negative (−), positive (+), strongly positive (++), and very strongly positive (+++) is shown. Scale bar, 50 mm. (**C**) Concomitant expression of Gli1 with SOX2 and BMI1. The consecutive sections of PTC and anaplastic thyroid carcinoma (ATC) specimens were immunostained with antibodies against Gli1, BMI1, and SOX2. Scale bar, 100 mm. (**D**) The signals of Gli1, SOX2, and BMI1 staining in the images taken from the consecutive sections of 46 PTC specimens were quantified by using an Imageproplus software. The arbitrary units of the staining signals were plotted in dot graphs.

Finally, we conducted immunohistochemistry staining to detect the expression of BMI1 and SOX2 in thyroid cancer. BMI1 (Figure 6A) and SOX2 (Figure 6B) signals were not present in normal thyroid follicular epithelial cells. Among 22 PTC sections with clearly identifiable normal thyroid follicular epithelial cells, BMI1 and SOX2 were all stained negative or with only very weak signals. Also shown in Figure 6A,B are PTCs graded negative (−), weak (+), moderate (++), and strong (+++). BMI1 and SOX2 signals were present mainly in the cytoplasm but also in the nucleus in some specimens. Normal rabbit IgG included as a negative control on a PTC specimen did not give any signals. We found that 46 of 64 PTC (73%) and all 5 ATCs were BMI1-positive; 45 of 64 PTCs (70%) and all 5 ATCs and were SOX2-positive. BMI1 and SOX2 expression did not correlate with the age and gender of the patients and were not associated with tumor types, stage, metastasis (local cervical lymph node invasion and distal metastasis), and BRAF mutations (Table 2). 

The Shh pathway plays a pivotal role in regulating cancer stem cell self-renewal [43]. Here we investigated if SOX2 and BMI1 expression correlated with Gli1 levels. As shown in Figure 6C, Gli1, BMI1, and SOX2 were all negative in the consecutive sections of a PTC specimen (left column) or positive in the consecutive sections of a PTC (middle column) and ATC (right column). Among all tumor samples, the overall accordance rates, which combine the results of Gli1-negative and Gli1-positive samples, between Gli1 and BMI1 or SOX2 expression were 85% and 84%, respectively (Table 3). When the intensity of Gli1, BMI1, and SOX2 signals was quantified and statistically analyzed, it was found that the Gli1 levels positively correlated with the levels of BMI1 and SOX2 (Figure 6D).

## 3. Discussion

Our present study focused on the expression and regulation of SOX2 and BMI1 by the Shh pathway in thyroid cancer. We made several important findings, including: 1) BMI1 and SOX2 were highly expressed in papillary and anaplastic thyroid cancers and correlated with Gli1 expression; 2) inhibition of the Shh pathway by two specific inhibitors or by siRNA suppressed SOX2 and BMI1 expression in vitro and in vivo, whereas Gli1 and Snail overexpression led to increased BMI1 and SOX2 expression; 3) Gli1, BMI1, and SOX2 levels were significantly higher in ALDH-positive than in ALDH-negative cells; 4) ALDH-positive CSCs had significantly higher tumorigenic potential than bulk tumor cells; 5) GANT61 suppressed thyroid CSC-derived tumor initiation and growth in a xenograft mouse model. Our study suggests that BMI1 and SOX2, two CSC-related transcription factors overexpressed in thyroid neoplasms, are regulated by the Shh pathway at a post-transcriptional and transcriptional level, respectively; and that targeting the Shh pathway could be a novel strategy for treating thyroid cancer. 

The Shh pathway stimulates CSC self-renewal of a variety of cancers by inducing the expression of several stemness-related genes, including c-MYC, BMI1, Nanog, SOX2, and OCT4 [44]. Truncated GLI1 activates the metastasis-initiating cancer stem cells and astrocytes in the breast cancer microenvironment and promotes its metastasis to the brain [45]. Our prior study demonstrated that inhibition of the Shh pathway leads to the suppression of thyrosphere formation and decreases the percent of ALDH-positive CSCs in thyroid cancer cell lines [22]. Here we provide further evidence that the inhibitors of the Shh pathway as well as Shh and Gli1 siRNA downregulated BMI1 and SOX2 expression. Based on the established role of BMI1 and SOX2 in stem cell self-renewal [24,33], we speculate that stimulation of thyroid CSC self-renewal by the Shh pathway is likely mediated through increased BMI1 and SOX2 expression. However, it should be noted that this supposition needs to be experimentally proven before any conclusions could be drawn. 

Emerging evidence suggests that Snail, a transcriptional factor induced by Gli1, not only regulates EMT induction, but also promotes CSC self-renewal [46,47,48]. For example, Snail overexpression promotes the development of the CD44^hi^CD24^lo^ population in immortalized mammary epithelial cells [49] and renders human squamous cell carcinoma with stem cell-like properties [50]. Snail-transgenic mice develop spontaneous thyroid cancer and have increased thyroid cancer incidence after irradiation [51]. We and others have shown that Snail is upregulated in thyroid cancer by activation of the Shh pathway and plays an important role in maintaining thyroid CSC self-renewal [6,22]. Our present study demonstrates that Snail siRNA downregulated the levels of BMI1 and SOX2 proteins; whereas Snail overexpression increased BMI1 and SOX2 expression. These observations collectively suggest that the Shh pathway may regulate SOX2 and BMI1 expression in part by Snail. 

BMI1 plays an important role in stem cell self-renewal, EMT, chemoresistance, and cell proliferation [24]. BMI1 mediates the Shh pathway-activated mammosphere formation in breast cancer [44,52]. Activation of the Shh pathway leads to increased BMI1 expression in medulloblastoma and breast cancer [44,53]. Using a chromatin immunoprecipitation assay, Wang et al. reported that Gli1 directly binds the promoter of BMI1 in medulloblastoma [53]. However, cyclopamine does not suppress BMI1 mRNA levels, Gli1 overexpression only marginally increases BMI1 mRNA levels in Daoy glioma spheres [53]. Our present study shows that BMI1 RNA levels were not affected by GANT61 or cyclopamine nor by Gli1 siRNA or Gli1 overexpression. In addition, the inhibitors of the Shh pathway also inhibited the expression of Gli1 and SOX2 much better than BMI1 (Figure 1). IHC analysis revealed that the correlation between BMI1 and Gli1 expression was lower than that between SOX2 and Gli1 (Figure 6D). These observations collectively suggested that BMI1 is post-transcriptionally and indirectly regulated by the Shh pathway. Several studies have shown that Gli1 regulates BMI1 expression indirectly by miR-128 [54,55,56]. Interestingly, Fu et al. [57] reported that the SMO inhibitor Erismodegib decreases BMI1 levels in glioblastoma cells by inducing miR-128 expression. Qian et al. [58] reported that loss of Snail expression leads to increased miR-128 expression and subsequently decreased Bmi1 expression. Our unpublished data shows that indeed miR-128-2 significantly decreased Bmi1 expression. However, inhibition of the Shh pathway did not significantly change the levels of miR-128. Therefore, it is not clear if Bmi1 is post-translationally regulated by ubiquitination (Figure S3). 

SOX2 is another stemness-related transcription factor regulated by the Shh pathway. Gli1 is responsible for increased SOX2 expression in cholangiocarcinoma under hypoxia conditions and promotes cancer cell stemness, epithelial-to-mesenchymal transition and invasion [59]. SOX2 is highly expressed in 7 ATC specimens [39] and is expressed at higher levels in thyrospheres than in adherent SW1736 cells [60] and in ALDH^+^ than in ALDH^-^ thyroid cancer cell lines [61]. Consistent with these observations, we found that SOX2 levels were significantly higher in ALDH-positive than ALDH-negative SW1736 cells. In addition, we found that SOX2 was also highly expressed in ATCs and in PTCs. We further showed that inhibition of the Shh pathway led to decreased SOX2 mRNA and promoter activity, suggesting that SOX2 expression is regulated by the Shh pathway at a transcriptional level. In support of this notion, two prior studies showed that Gli1 induces SOX2 expression in melanoma and pancreatic cancer through a cis-element in the SOX2 promoter [62,63]. SOX2, in addition to being transcriptionally regulated by Gli1, is post-transcriptionally regulated in gastric cancer by AKT activation in a ubiquitin-dependent manner [64]. Our prior study showed that the Shh pathway induces AKT phosphorylation in thyroid cancer [23]. Our present study shows that Snail, a downstream transcription factor of Gli1, also induced SOX2 expression. We speculate that SOX2 expression is regulated by Snail at a post-transcriptional level. Interestingly, a recent study by Zhang et al. [65] showed that circular RNA circ_0005273 regulates SOX2 expression and promotes thyroid carcinoma progression. Thus, SOX2 expression in thyroid cancer could be regulated by multiple mechanisms [33].

BMI1 and SOX2 have been sought as novel molecular targets for preventing tumor metastasis and recurrence [24,66,67,68,69]. PTC-028, a specific inhibitor of BMI1, inhibits CSC self-renewal of medulloblastoma in vitro, reduces tumor burden in both local and metastatic compartments, and suppresses the tumor initiation ability of recurrent medulloblastoma in vivo [70]. Mesenchymal glioma stem cells are sensitive to a BMI1 inhibitor [71]. BMI1 inhibitors impair tumor growth and suppress glioma and lung cancer tumorigenesis [71,72]. Our present study shows that transient treatment with GANT61 suppressed the growth of thyroid tumor xenografts derived from ALDH-positive SW1736 cells. GANT61 weakly suppressed the growth of tumor xenografts derived from unsorted SW1736 cells even though the mice were continually treated with GANT61 until the end of the experiment. GANT61 treatment significantly decreased BMI1 and SOX2 expression in vivo in tumor xenografts (Figure 5D). We speculate that GANT61 suppresses tumor development largely by inhibiting the growth of thyroid CSC-driven tumors. 

GANT61 is a leading Gli1 inhibitor and has been widely used as a novel anticancer agent in preclinical studies [73]. Other Gli1 inhibitors such as ATO or HPI are either not specific or poorly characterized [73]. In addition, Gli1 can be cross-activated by other signaling pathways, Smo inhibitors often develop drug resistance due to Gli1 cross-activation [43,73]. We speculate that the SMO inhibitor such as Vismodegib or itraconazole may not have better therapeutic effects than GANT61. Therefore, we only used one inhibitor of the Shh pathway, GANT61, in our in vivo experiments. Since anaplastic thyroid cancer is highly metastatic, future studies should also pay attention to the effect of GANT61 and Smo inhibitors on tumor metastasis. Moreover, since BMI1 appears to be a crucial transcription factor regulated by Gli1, having shown that BMI1 inhibitors can control thyroid CSC self-renewal, BMI1 inhibitors should be further evaluated for their inhibitory activity on tumor growth and metastasis. 

We are aware of several weaknesses in our current study. First, we did not confirm the ability of Gli1 to directly bind the SOX2 promoter. Second, we did not conduct a detailed analysis on how Snail is involved in regulating the expression of BMI1 and SOX2. Third, though it is well established that BMI1 and SOX2 play an important role in regulating stem cell self-renewal in a variety of malignancies, we did not further investigate if this is the case in thyroid cancer. Thus, it remains elusive if the Shh pathway regulates thyroid CSC self-renewal by BMI1 and SOX2. Nevertheless, our study provides evidence that activation of the Shh pathway plays an important role in regulating SOX2 and BMI1 expression in thyroid cancer. Targeting the Shh pathway delayed thyroid CSC-driven tumor growth. Our study grants new insights into the mechanisms of increased BMI1 and SOX2 expression in thyroid cancer and highlights the potential of the Shh pathway inhibitors as anticancer agents for preventing tumor recurrence. 

## 4. Materials and Methods

### 4.1. Reagents 

Cyclopamine was purchased from Selleck Chemicals LLC (Shanghai, China) and dissolved in dimethyl sulfoxide (DMSO). GANT61 was purchased from Medkoo Biosciences (Morrisville, NC, USA) and dissolved in 100% ethanol. For in vivo experiments, GANT61 was diluted in corn oil. Antibodies against Shh (Catalog # 2207), Gli1 (Catalog # 2643), Snail (Catalog # 3895), Bmi1 (Catalog # 6964) were purchased from Cell Signaling Technology, Inc. (Danvers, MA, USA). SOX2 (Catalog #AM2048) was purchased from ABGENT (San Diego, CA, USA). ALDH antibody (Catalog # 611194) was purchased from Beckton Dickson Company (Franklin Lakes, NJ, USA). Antibody against β-actin (Catalog # sc-47778) and anti-Gli1 antibody (Catalog # sc-515751) used in immunofluorescence staining (Figure 5) was obtained from Santa Cruz Biotechnology Inc. (San Diego, CA, USA). Normal rabbit IgG (Catalog #15006) and normal mouse IgG (Catalog # 12-371) were purchased from Sigma-Aldrich Inc. (St. Louis, MO, USA). Shh (Catalog # L-006036-00), Gli1 (Catalog # SR301820C), and Snail (Catalog # L-010847-01) siRNA ON-TARGETplus SMARTpools were synthesized by Dharmacon and purchased from Fisher Scientific (Pittsburg, PA, USA). The ALDEFLUOER kit used for sorting thyroid CSCs was purchased from STEMCELL Technologies, Inc. (Vancouver, BC, Canada). Luciferase substrate was purchased from TransGen Biotech (Beijing, China). 

### 4.2. Cell Lines and Plasmid DNA

Two ATC cell lines, KAT-18 and SW1736, were kindly provided by Dr. Kenneth B. Ain [74], authenticated, and reported earlier [22,42]. KAT-18 cells were used between 40 and 60 passages; SW1736 cells were used between 26–45 passages. Both cell lines were grown in complete RPMI 1640 media containing 10% fetal bovine serum. An 8-copy Gli1 binding site-driven luciferase reporter gene (8 × 3′Gli-BS-Luc) and a pcDNA3.1 encoding a human Gli1 (pcDNA/Gli1) were kindly provided by Dr. Tsutomu Kume (Northwestern University Feinberg School of Medicine, Chicago, IL, USA). The pcDNA/Snail-HA expression vector (#31697) was purchased from Addgene (Watertown, MA, USA). The SOX2 promoter was cloned into the pGL3/Basic vector by ligation of a Kpn I and Hind III-digested 1041-bp PCR product and designated as pGL3/SOX2-Luc. The primers used to amplify the human SOX2 promoter were 5′-ATGCTAGGTACCGGCCAAAGAGCTGAGTTGGA-3′ and 5′-CGCAGCAAGC-TTGAGGCAAACTGGAATCAGGATC-3′. 

### 4.3. Tumor Specimens and Patient Information 

After approval by the institutional review board of Yangzhou University, the paraffin-embedded tumor blocks from patients with thyroid neoplasms were retrieved and used for immunohistochemical staining. Sixty-nine thyroid cancer specimens, all with adequate clinical and pathological information, were studied for the expression of SOX2, BMI1, and Gli1 expression. These include 64 papillary carcinomas and 5 anaplastic carcinomas. Normal thyroid follicular cells in the transitional zone next to tumor cells in a total of 22 specimens were also graded for Gli1, BMI1, and SOX2 expression. The correlation between Gli1 expression and the levels of SOX2 and BMI1 was determined by scanning the images taken from consecutive sections of 46 PTC specimens using Image-Pro Plus software Version 7.0 (Rockville, MD, USA). The arbitrary units of the staining signals were plotted in dot graphs. 

### 4.4. Immunohistochemical (IHC) Staining of BMI1, SOX2, and Gli1 in Thyroid Tumor Tissues

The sections of paraffin-embedded tissue blocks were stained for BMI1, SOX2, and Gli1 according to our prior publication [42]. IHC staining was graded as negative (−), no signal at all; positive (+), with a signal in >20% of tumor cells; strongly positive (++), with a strong signal in more than 50% of tumor cells; very strongly positive (+++), with a very strong signal in >80% of tumor cells. Three random fields per slide in tumor zones were graded by two investigators (X. Xu and Y. Lu) in a blinded fashion. 

### 4.5. RT-PCR

Total cellular RNA was extracted from cell lines with TRIzol (Sangon Biotech, Shanghai, China.) and quantified by UV absorption. RNA integrity was verified by electrophoresis. Reverse transcription of RNA was performed using PrimeScript RT Master Mix (TAKARA, Bio, Inc. Kusatsu, Shiga, Japan) according to the manufacturer’s protocol. The cDNA was subjected to quantitative real-time PCR using a CFX Connect Real-time system (Bio-Rad, Hercules, CA, USA) and SYBR Green Supermix (Bio-Rad, Hercules, CA, USA). Amplification of human glyceraldehyde 3-phosphate dehydrogenase (GAPDH) was included as an internal control. The primers for amplifying Gli1 were 5′-TTCCTACCAGAGTCCCAAGT-3′ (forward) and 5′-CCCTATGTGAAGCCCTATTT-3′ (reverse); The primers for amplifying GAPDH were 5′-TGAAGGTCGGAGTCAACGGATTTGGTC-3′ (forward) and 5′-ATGGACTGTGG TCATGAGTCCTTCCACG-3′ (reverse). The primers used to amplify SOX2 were 5′-GGAGAGTAAGAAACAGCATGGA-3′ (forward) and 5′-GTGGATGGGATTGGTGTTCT-3′ (reverse) (477-bp). The primers for amplifying BMI1 were 5’-GTGCTTTGTGGAGGGTACTT-3′ (forward) and 5′-GTCTCCAGGTAACGAACAATACA-3′ (reverse). Of note, the forward and reverse primers for each gene locate in different exons, thus excluding the possibility of amplifying a PCR product from contaminated genomic DNA. The PCR reaction was set with an initial denaturation of 30 s at 95 °C and subsequent 40 cycles of denaturation for 5 s at 95 °C, annealing for 30 s at 60 °C, and extension for 15 s at 72 °C. All expression levels were normalized to GAPDH levels in the same sample. Fold change was calculated by the ΔΔCT method. Percent expression was calculated as the ratio of the normalized value of each sample to that of the corresponding untreated control sample. All real-time PCR analyses were performed in triplicate.

### 4.6. Western Blot

SW1736 and KAT-18 cells seeded in 6-well plates were treated with the indicated concentrations of cyclopamine or GANT61 for 48 h or transfected with Shh, Gli1, and Snail siRNA or with Gli1 and Snail expression vectors according to the manufacturer’s instruction. Cell lysates were prepared as described [22] and analyzed for the expression of Shh, Gli1, Snail, ALDH, BMI1, SOX2. For loading controls, β-actin was detected by a mouse monoclonal antibody. The density of protein blots was analyzed by using NIH Image-J software. The levels of the protein of interest relative to the β-actin controls were analyzed by the formula (the reading of the band of interest − the reading of a blank area) / (the reading of β-actin band − the reading of a blank area). The results were presented as the mean ± standard deviation (SD) of three experiments in bar graphs. All original western blot images could be viewed in File S1.

### 4.7. Luciferase Reporter Assay

KAT-18 and SW1736 cells were transfected with an 8-copy Gli1 binding site-driven luciferase reporter gene (8 × 3′Gli-BS-Luc) by using TurboFect Transfection Reagent (Thermo Fisher Scientific Inc., Waltham, MA, USA) following the manufacturer’s instructions. An internal control plasmid (the β-actin promoter-driven Renilla luciferase reporter) was included in the transfection mixture. Luciferase reporter gene without a promoter (pGL3/Basic) was included as a negative control. After incubation for 48 h, the cells were harvested and analyzed for luciferase activity by using the luciferin substrate and reading in a TECAN plate reader (Phenix Research Products, Hayward, CA, USA). The relative light unit in each sample was normalized against the β-actin promoter-driven Renilla luciferase control. The means ± standard deviations (SD) of the data in triplicate from one experiment are presented. The experiments were repeated at least twice with similar results.

### 4.8. Gli1 and Snail Knockdown

KAT-18 and SW1736 cells seeded in a 6-well plate were transfected with Snail siRNA by using Lipofectamine RNAiMAX (Invitrogen Life Technologies, Grand Island, NY, USA) according to the manufacturer’s instruction. After incubation for 48 h, cell lysates were analyzed by Gli1, Snail, BMI1, and SOX2 expression by Western blot with their specific antibodies. 

### 4.9. Sorting of ALDH-Positive Cells

Single-cell suspensions of SW1736 were prepared and analyzed for ALDH by using an ALDEFLUOR kit as previously reported [22]. Diethylaminobenzaldehyde (DEAB), an ALDH-specific inhibitor, was added to the aliquot of SW1736 cells before staining as a background control. Cell lysates from ALDH-positive and ALDH-negative populations were analyzed by Western blot for BMI1, SOX2, ALDH, Gli1, and Actin expression. ALDH-positive cells were also used in the in vivo experiments. 

### 4.10. Xenograft Thyroid Tumor Model

The study was carried out in compliance with the recommendations in the Guide for the Care and Use of Laboratory Animals of the National Institutes of Health. The protocol was approved by the Institutional Animal Care and Use Committee of Yangzhou University (protocol code 2018030301 and date of approval 3 March 2018). Female NCG mice were purchased from Beijing Vital River Laboratory Animal Technology Co., Ltd., Beijing, China. Mice (5–6-weeks-old; 5 mice/group) were injected subcutaneously with the indicated numbers of ALDH-positive cells (5 × 10^2^, 5 × 10^3^, or 5 × 10^4^ cells/mouse) or with unsorted bulk SW1736 cells (5 × 10^3^, 5 × 10^4^, or 5 × 10^5^ cells/mouse) into the right flank of back skin. The mice were observed twice weekly for tumor occurrence for 3 months. To determine the anticancer effect of GANT61 against thyroid tumors derived from thyroid CSCs, ALDH-positive SW1736 cells (5 × 10^4^ cells/mouse) were injected subcutaneously into NCG mice. One week later, the mice were treated with corn oil as the vehicle (ethanol:corn oil, 1:4 vov:vol) or GANT61 (35 mg/kg/day) every other day for two weeks by intraperitoneal injection. Tumor volumes were measured twice a week with a caliper and calculated based on the formula length × width^2^ × π ÷ 6. Mice were sacrificed on day 70 after tumor cell injection. Tumors were collected and weighed. To determine the anticancer effect of GANT61 against thyroid tumors derived from bulk tumor cells, unsorted SW1736 cells (1 × 10^6^ cells/mouse) were injected subcutaneously into the mice. Three days later, mice were treated with the corn oil vehicle or GANT61 (35 mg/kg/day) (8 mice per group) every other day for 3 weeks by intraperitoneal injection. Mice were sacrificed on day 28 after tumor cell injection. The tumor tissues were collected and fixed in 10% formalin and then embedded in paraffin within 48 h. The sections of paraffin-embedded xenograft tumor blocks were deparaffinized, rehydrated, and stained with hematoxylin and eosin (H&E). 

### 4.11. Immunofluorescence Staining

The sections of frozen tissue blocks were fixed in 4% paraformaldehyde, blocked with 5% bovine serum albumin (BSA) blocking buffer in PBS for 30 min at room temperature, and then incubated with rabbit antibodies against Gli1 (Catalog # sc-515751, Santa Cruz Biotechnology, Inc., 1:50), SOX2 (Catalog #AM2048, Cell Signaling Inc., 1:200), and BMI1 (Cell Signaling Inc., Catalog # 6964) (1:200) in PBS overnight at 4 °C. Following incubation, the sections were stained with Alexa Fluor-488-conjugated anti-rabbit IgG (AB_2338064) or anti-mouse IgG (AB_2338840) (Jackson ImmunoResearch Laboratories Inc., West Grove, PA, USA) (1:100) or for 1 h at room temperature. Normal rabbit and mouse IgG were used as the negative controls. Finally, the sections were stained with 10 µM DAPI (4′,6-diamidino-2-phenylindole) for 5 min. After washing the cells in PBS, fluorescent images were captured under a Nikon fluorescence microscope. 

### 4.12. Statistical Analysis Bmi1 

The differences in mRNA levels, luciferase activity, and density of Western blots between different treatment groups were statistically analyzed by using an unpaired Student’s *t* test. The differences in tumor occurrence rates were statistically analyzed by the *X^2^* test. The correlation between Gli1 and BMI1 and SOX2 expression was analyzed by the *X^2^* test (Table 3) and the regression test (Figure 6D). The differences in tumor volumes were statistically analyzed by using the one-way repeated measure ANOVA. The *p* value of <0.05 was considered statistically significant. All statistical tests was performed with SigmaPlot 11 software (Systat Software, Inc, San Jose, CA, USA).

## 5. Conclusions

In conclusion, our study shows that *SOX2* and *BMI1*, two cancer cell stemness-related genes, are regulated by the Shh pathway in thyroid cancer; Gli1 expression correlates with the levels of SOX2 and BMI1 in the PTC specimens; Inhibition of the Shh pathway leads to the retardation of thyroid CSC-driven tumor growth but does not significantly affect the growth of tumor xenografts derived from bulk thyroid tumor cells; ALDH-positive thyroid CSCs express high levels of the *SOX2* and *BMI* genes and are highly tumorigenic. The Shh pathway inhibitors could be potentially useful for preventing tumor recurrence.

## Figures and Tables

**Table 1:** Comparison of the ability of aldehyde dehydrogenase (ALDH)-positive and ALDH-negative. SW1736 to develop tumors in immunodeficient NCG (NOD/ShiLtJGpt-*Prkdc*^em26^Il2rg^em26^/Gpt) mice.

Number of ALDH^+^ Cells	Number of Mice with Tumor	Number of ALDH^-^ Cells	Number of Mice with Tumor
5 × 10^2^	1/5	5 × 10^3^	0/5
5 × 10^3^	5/5	5 × 10^4^	4/5
5 × 10^4^	5/5	5 × 10^5^	5/5

**Table 2:** BMI1 and SOX2 expression in thyroid papillary carcinomas.

Invasion & Metastasis	Number	BMI1^+^ (%)	SOX2^+^ (%)	*p* Value
Yes	20	16 (80)	15 (75)	>0.05
No	44	30 (68)	30 (68)	
Age				
14–54	47	32 (68)	31 (66)	>0.05
≥55	17	15 (88)	16 (94)	
Sex				
Male	21	15 (71)	15 (71)	>0.05
Female	43	31 (72)	30 (70)	
Tumor stage				
T1	15	9 (60)	9 (60)	>0.05
T2	33	25 (76)	25 (76)	
T3	10	7 (70)	6 (60)	
T4	6	5 (83)	5 (83)	
BRAF mutation				
Yes	20	15 (75)	15 (75)	>0.05
No	44	31 (70)	30 (68)	

**Table 3:** Concomitant expression between Gli1 and BMI1 or SOX2.

Tumor Type	Number	BMI1^+^ (%)	*p* Value	S0X2^+^ (%)	*p* Value
**ATC**					
Gli1^+^	5	5 (100%)		5 (100%)	
Gli1^−^	0	0 (0%)	<0.01	0 (0%)	<0.01
**PTC**					
Gli1^+^	50	46 (93%)		45 (90%)	
Gli1^−^	14	6 (43%)	<0.01	6(43%)	<0.01

## Data Availability

The data presented in this study are available on request from the corresponding author. The data are not publicly available due to ethical issues.

## Supplementary Materials

The following are available online at https://www.mdpi.com/2072-6694/13/3/418/s1, Figure S1: Cyclopamine inhibits BMI1 and SOX2 expression in WRO82 cells., Figure S2: Immunofluorescence staining with negative control antibodies., Figure S3: miR-128 suppresses thyroid cancer stem cell self-renewal., File S1: Original Western blot images.

## References

[1] Kondo T., Ezzat S., Asa S.L. (2006). Pathogenetic mechanisms in thyroid follicular-cell neoplasia. Nat. Rev. Cancer.

[2] Fagin J.A., Wells S.A. (2016). Biologic and Clinical Perspectives on Thyroid Cancer. N. Engl. J. Med..

[3] Matrone A., Campopiano M.C., Nervo A., Sapuppo G., Tavarelli M., De Leo S. (2020). Differentiated Thyroid Cancer, From Active Surveillance to Advanced Therapy: Toward a Personalized Medicine. Front. Endocrinol..

[4] Cabanillas M.E., McFadden D.G., Durante C. (2016). Thyroid cancer. Lancet.

[5] Todaro M., Iovino F., Eterno V., Cammareri P., Gambara G., Espina V., Gulotta G., Dieli F., Giordano S., De Maria R. (2010). Tumorigenic and Metastatic Activity of Human Thyroid Cancer Stem Cells. Cancer Res..

[6] Ma R., Minsky N., Morshed S.A., Davies T.F. (2014). Stemness in Human Thyroid Cancers and Derived Cell Lines: The Role of Asymmetrically Dividing Cancer Stem Cells Resistant to Chemotherapy. J. Clin. Endocrinol. Metab..

[7] Altaba A.R., Sánchez P., Dahmane N. (2002). Gli and hedgehog in cancer: Tumours, embryos and stem cells. Nat. Rev. Cancer.

[8] Hooper J.E., Scott M.P. (2005). Communicating with Hedgehogs. Nat. Rev. Mol. Cell Biol..

[9] Taipale J., Beachy P.A. (2001). The Hedgehog and Wnt signalling pathways in cancer. Nat. Cell Biol..

[10] van den Brink G.R. (2007). Hedgehog signaling in development and homeostasis of the gastrointestinal tract. Physiol. Rev..

[11] Liu A., Wang B., Niswander L.A. (2005). Mouse intraflagellar transport proteins regulate both the activator and repressor functions of Gli transcription factors. Development.

[12] Kim J., Kato M., Beachy P.A. (2009). Gli2 trafficking links Hedgehog-dependent activation of Smoothened in the primary cilium to transcriptional activation in the nucleus. Proc. Natl. Acad. Sci. USA.

[13] Merchant A.A., Matsui W. (2010). Targeting Hedgehog—A cancer stem cell pathway. Clin. Cancer Res..

[14] Woodward W.A., Chen M.S., Behbod F., Rosen J.M. (2005). On mammary stem cells. J. Cell Sci..

[15] Kasper M., Jaks V., Fiaschi M., Toftgård R. (2009). Hedgehog signalling in breast cancer. Carcinog.

[16] Satheesha S., Manzella G., Bovay A., A Casanova E., Bode P.K., Belle R., Feuchtgruber S., Jaaks P., Dogan N., Koscielniak E. (2015). Targeting hedgehog signaling reduces self-renewal in embryonal rhabdomyosarcoma. Oncogene.

[17] Peacock C.D., Wang Q., Gesell G.S., Corcoran-Schwartz I.M., Jones E., Kim J., Devereux W.L., Rhodes J.T., Huff C.A., Beachy P.A. (2007). Hedgehog signaling maintains a tumor stem cell compartment in multiple myeloma. Proc. Natl. Acad. Sci. USA.

[18] Naka K., Hoshii T., Hirao A. (2010). Novel therapeutic approach to eradicate tyrosine kinase inhibitor resistant chronic myeloid leukemia stem cells. Cancer Sci..

[19] Zhao C., Chen A., Jamieson C.H., Fereshteh M.P., Abrahamsson A., Blum J., Kwon H.Y., Kim J., Chute J.P., A Rizzieri D. (2009). Hedgehog signalling is essential for maintenance of cancer stem cells in myeloid leukaemia. Nat. Cell Biol..

[20] Nicolis S.K. (2007). Cancer stem cells and “stemness” genes in neuro-oncology. Neurobiol. Dis..

[21] Xu Q., Yuan X., Liu G., Black K.L., Yu J.S. (2008). Hedgehog Signaling Regulates Brain Tumor-Initiating Cell Proliferation and Portends Shorter Survival for Patients with PTEN-Coexpressing Glioblastomas. STEM CELLS.

[22] Heiden K.B., Williamson A.J., Doscas M.E., Ye J., Wang Y., Liu D., Xing M., Prinz R.A., Xu X. (2014). The Sonic Hedgehog Signaling Pathway Maintains the Cancer Stem Cell Self-Renewal of Anaplastic Thyroid Cancer by Inducing Snail Expression. J. Clin. Endocrinol. Metab..

[23] Williamson A.J., Doscas M.E., Ye J., Heiden K.B., Xing M., Li Y., Prinz R.A., Xu X. (2016). The sonic hedgehog signaling pathway stimulates anaplastic thyroid cancer cell motility and invasiveness by activating Akt and c-Met. Oncotarget.

[24] Siddique H.R., Saleem M. (2012). Role of BMI1, a stem cell factor, in cancer recurrence and chemoresistance: Preclinical and clinical evidences. Stem Cells.

[25] Song L., Li J., Liao W., Feng Y., Yu C., Hu L., Kong Q., Xu L., Zhang X., Liu W. (2009). The polycomb group protein Bmi-1 represses the tumor suppressor PTEN and induces epithelial-mesenchymal transition in human nasopharyngeal epithelial cells. J. Clin. Investig..

[26] Chiba T., Miyagi S., Saraya A., Aoki R., Seki A., Morita Y., Yonemitsu Y., Yokosuka O., Taniguchi H., Nakauchi H. (2008). The Polycomb Gene Product BMI1 Contributes to the Maintenance of Tumor-Initiating Side Population Cells in Hepatocellular Carcinoma. Cancer Res..

[27] Chiba T., Seki A., Aoki R., Ichikawa H., Negishi M., Miyagi S., Oguro H., Saraya A., Kamiya A., Nakauchi H. (2010). Bmi1promotes hepatic stem cell expansion and tumorigenicity in bothInk4a/Arf-dependent and -independent manners in Mice. Hepatology.

[28] Proctor E., Waghray M., Lee C.J., Heidt D.G., Yalamanchili M., Li C., Bednar F., Simeone D.M. (2013). Bmi1 enhances tumorigenicity and cancer stem cell function in pancreatic adenocarcinoma. PLoS ONE.

[29] Yu C.-C., Lo W.-L., Chen Y.-W., Huang P.-I., Hsu H.-S., Tseng L.-M., Hung S.-C., Kao S.-Y., Chang C.-J., Chiou S.-H. (2010). Bmi-1 Regulates Snail Expression and Promotes Metastasis Ability in Head and Neck Squamous Cancer-Derived ALDH1 Positive Cells. J. Oncol..

[30] Chou C.-H., Yang N.-K., Liu T.-Y., Tai S.-K., Hsu D.S.-S., Chen Y.-W., Chen Y.-J., Chang C.-C., Tzeng C.H., Yang M.-H. (2013). Chromosome Instability Modulated by BMI1–AURKA Signaling Drives Progression in Head and Neck Cancer. Cancer Res..

[31] Yang M.-H., Hsu D.S.-S., Wang H.-W., Wang H.-J., Lan H.-Y., Yang W.-H., Huang C.-H., Kao S.-Y., Tzeng C.-H., Tai S.-K. (2010). Bmi1 is essential in Twist1-induced epithelial–mesenchymal transition. Nat. Cell Biol..

[32] Ferretti R., Bhutkar A., McNamara M.C., Lees J.A. (2015). BMI1 induces an invasive signature in melanoma that promotes metastasis and chemoresistance. Genes Dev..

[33] Schaefer T., Lengerke C. (2020). SOX2 protein biochemistry in stemness, reprogramming, and cancer: The PI3K/AKT/SOX2 axis and beyond. Oncogene.

[34] Garros-Regulez L., Garcia I., Carrasco-Garcia E., Lantero A., Aldaz P., Moreno-Cugnon L., Arrizabalaga O., Undabeitia J., Torres-Bayona S., Villanua J. (2016). Targeting SOX2 as a Therapeutic Strategy in Glioblastoma. Front. Oncol..

[35] Rodriguez-Pinilla S.M., Sarrio D., Moreno-Bueno G., Rodriguez-Gil Y., A Martinez M., Hernandez L., Hardisson D., Reis-Filho J.S., Palacios J. (2007). Sox2: A possible driver of the basal-like phenotype in sporadic breast cancer. Mod. Pathol..

[36] Gure A.O., Stockert E., Scanlan M.J., Keresztes R.S., Jager D., Altorki N.K., Old L.J., Chen Y.T. (2000). Serological identification of embryonic neural proteins as highly immunogenic tumor antigens in small cell lung cancer. Proc. Natl. Acad. Sci. USA.

[37] Gangemi R.M.R., Griffero F., Marubbi D., Perera M., Capra M.C., Malatesta P., Ravetti G.L., Zona G.L., Daga A., Corte G. (2009). SOX2Silencing in Glioblastoma Tumor-Initiating Cells Causes Stop of Proliferation and Loss of Tumorigenicity. STEM CELLS.

[38] Zhu S., Zhao D., Li C., Li Q., Jiang W., Liu Q., Wang R., Fazli L., Li Y., Zhang L. (2020). BMI1 is directly regulated by androgen receptor to promote castration-resistance in prostate cancer. Oncogene.

[39] Carina V., Zito G., Pizzolanti G., Richiusa P., Criscimanna A., Rodolico V., Tomasello L., Pitrone M., Arancio W., Giordano C. (2013). Multiple Pluripotent Stem Cell Markers in Human Anaplastic Thyroid Cancer: The Putative Upstream Role of SOX2. Thyroid..

[40] Ok C.Y., Singh R.R., Vega F. (2012). Aberrant activation of the hedgehog signaling pathway in malignant hematological neoplasms. Am. J. Pathol..

[41] Lu L., Chen Z., Lin X., Tian L., Su Q., An P., Li W., Wu Y., Du J., Shan H. (2020). Inhibition of BRD4 suppresses the malignancy of breast cancer cells via regulation of Snail. Cell Death Differ..

[42] Xu X., Ding H., Rao G., Arora S., Saclarides C.P., Esparaz J., Gattuso P., Solorzano C.C., A Prinz R. (2012). Activation of the Sonic Hedgehog pathway in thyroid neoplasms and its potential role in tumor cell proliferation. Endocrine-Related Cancer.

[43] Xu X., Lu Y., Li Y., Prinz R.A. (2017). Sonic Hedgehog Signaling in Thyroid Cancer. Front. Endocrinol..

[44] Liu S., Dontu G., Mantle I.D., Patel S., Ahn N.-S., Jackson K.W., Suri P., Wicha M.S. (2006). Hedgehog Signaling and Bmi-1 Regulate Self-renewal of Normal and Malignant Human Mammary Stem Cells. Cancer Res..

[45] Sirkisoon S.R., Carpenter R.L., Rimkus T., Doheny D., Zhu D., Aguayo N.R., Xing F., Chan M., Ruiz J., Metheny-Barlow L.J. (2020). TGLI1 transcription factor mediates breast cancer brain metastasis via activating metastasis-initiating cancer stem cells and astrocytes in the tumor microenvironment. Oncogene.

[46] Li X., Deng W., Lobo-Ruppert S.M., Ruppert J.M. (2007). Gli1 acts through Snail and E-cadherin to promote nuclear signaling by beta-catenin. Oncogene.

[47] Li X., Deng W., Nail C.D., Bailey S.K., Kraus M.H., Ruppert J.M., Lobo-Ruppert S.M. (2006). Snail induction is an early response to Gli1 that determines the efficiency of epithelial transformation. Oncogene.

[48] Baulida J., Garcia de Herreros A. (2015). Snail1-driven plasticity of epithelial and mesenchymal cells sustains cancer malignancy. Biochim. Biophys. Acta.

[49] Tam W.L., Lu H., Buikhuisen J., Soh B.S., Lim E., Reinhardt F., Wu Z.J., Krall J.A., Bierie B., Guo W. (2013). Protein Kinase C α Is a Central Signaling Node and Therapeutic Target for Breast Cancer Stem Cells. Cancer Cell.

[50] Zhu L.-F., Hu Y., Yang C.-C., Xu X.-H., Ning T.-Y., Wang Z.-L., Ye J.-H., Liu L.-K. (2012). Snail overexpression induces an epithelial to mesenchymal transition and cancer stem cell-like properties in SCC9 cells. Lab. Investig..

[51] Hardy R.G., Vicente-Dueñas C., González-Herrero I., Anderson C., Flores T., Hughes S., Tselepis C., Ross J.A., Sánchez-García I. (2007). Snail Family Transcription Factors Are Implicated in Thyroid Carcinogenesis. Am. J. Pathol..

[52] Sun Y., Wang Y.-S., Fan C., Gao P., Wang X., Wei G., Wei J. (2014). Estrogen promotes stemness and invasiveness of ER-positive breast cancer cells through Gli1 activation. Mol. Cancer.

[53] Wang X., Venugopal C., Manoranjan B., McFarlane N., O’Farrell E., Nolte S., Gunnarsson T., Hollenberg R., Kwiecien J., Northcott P. (2011). Sonic hedgehog regulates Bmi1 in human medulloblastoma brain tumor-initiating cells. Oncogene.

[54] Peruzzi P., Bronisz A., Nowicki M.O., Wang Y., Ogawa D., Price R., Nakano I., Kwon C.H., Hayes J., Lawler S.E. (2013). MicroRNA-128 coordinately targets Polycomb Repressor Complexes in glioma stem cells. Neuro Oncol..

[55] Zhu Y., Yu F., Jiao Y., Feng J., Tang W., Yao H., Gong C., Chen J., Su F., Zhang Y. (2011). Reduced miR-128 in Breast Tumor–Initiating Cells Induces Chemotherapeutic Resistance via Bmi-1 and ABCC5. Clin. Cancer Res..

[56] Godlewski J., Nowicki M.O., Bronisz A., Williams S., Otsuki A., Nuovo G., Raychaudhury A., Newton H.B., Chiocca E.A., Lawler S. (2008). Targeting of the Bmi-1 Oncogene/Stem Cell Renewal Factor by MicroRNA-128 Inhibits Glioma Proliferation and Self-Renewal. Cancer Res..

[57] Fu J., Rodova M., Nanta R., Meeker D., Van Veldhuizen P.J., Srivastava R.K., Shankar S. (2013). NPV-LDE-225 (Erismodegib) inhibits epithelial mesenchymal transition and self-renewal of glioblastoma initiating cells by regulating miR-21, miR-128, and miR-200. Neuro-Oncology.

[58] Qian P., Banerjee A., Wu Z., Zhang X., Wang H., Pandey V., Zhang W., Lv X., Tan S., Lobie P.E. (2012). Loss of SNAIL regulated miR-128-2 on chromosome 3p22.3 targets multiple stem cell factors to promote transformation of mammary epithelial cells. Cancer Res..

[59] Bhuria V., Xing J., Scholta T., Bui K.C., Nguyen M.L.T., Malek N.P., Bozko P., Plentz R.R. (2019). Hypoxia induced Sonic Hedgehog signaling regulates cancer stemness, epithelial-to-mesenchymal transition and invasion in cholangiocarcinoma. Exp. Cell Res..

[60] Malaguarnera R., Frasca F., Garozzo A., Gianì F., Pandini G., Vella V., Vigneri R., Belfiore A. (2011). Insulin Receptor Isoforms and Insulin-Like Growth Factor Receptor in Human Follicular Cell Precursors from Papillary Thyroid Cancer and Normal Thyroid. J. Clin. Endocrinol. Metab..

[61] Hardin H., Yu X.-M., Harrison A.D., Larrain C., Zhang R., Chen J., Chen H., Lloyd R.V. (2016). Generation of Novel Thyroid Cancer Stem-Like Cell Clones: Effects of Resveratrol and Valproic Acid. Am. J. Pathol..

[62] Santini R., Pietrobono S., Pandolfi S., Montagnani V., Damico M., Penachioni J.Y., Vinci M.C., Borgognoni L., Stecca B. (2014). SOX2 regulates self-renewal and tumorigenicity of human melanoma-initiating cells. Oncogene.

[63] Jia Y., Gu D., Wan J., Yu B., Zhang X., Chiorean E., Wang Y., Xie J. (2019). The role of GLI-SOX2 signaling axis for gemcitabine resistance in pancreatic cancer. Oncogene.

[64] Wang Z., Kang L., Zhang H., Huang Y., Fang L., Li M., Brown P.J., Arrowsmith C.H., Li J., Wong J. (2019). AKT drives SOX2 overexpression and cancer cell stemness in esophageal cancer by protecting SOX2 from UBR5-mediated degradation. Oncogene.

[65] Zhang W., Zhang H., Zhao X. (2020). circ_0005273 promotes thyroid carcinoma progression by SOX2 expression. Endocrine-Related Cancer.

[66] Stone L. (2016). Prostate cancer: Inhibiting initiation—Targeting BMI1 is effective. Nat. Rev. Urol..

[67] Giannone G., Attademo L., Scotto G., Genta S., Ghisoni E., Tuninetti V., Aglietta M., Pignata S., Valabrega G. (2019). Endometrial Cancer Stem Cells: Role, Characterization and Therapeutic Implications. Cancers.

[68] Grimm D., Bauer J., Wise P., Krüger M., Simonsen U., Wehland M., Infanger M., Corydon T.J. (2020). The role of SOX family members in solid tumours and metastasis. Semin. Cancer Biol..

[69] Hüser L., Novak D., Umansky V., Altevogt P., Utikal J. (2018). Targeting SOX2 in anticancer therapy. Expert Opin. Ther. Targets.

[70] Bakhshinyan D., Venugopal C., Adile A.A., Garg N., Manoranjan B., Hallett R., Wang X., Mahendram S., Vora P., Vijayakumar T. (2018). BMI1 is a therapeutic target in recurrent medulloblastoma. Oncogene.

[71] Jin X., Kim L.J.Y., Wu Q., Wallace L.C., Prager B.C., Sanvoranart T., Gimple R.C., Wang X., Mack S.C., Miller T.E. (2017). Targeting glioma stem cells through combined BMI1 and EZH2 inhibition. Nat. Med..

[72] Yong K.J., Basseres D.S., Welner R.S., Zhang W.C., Yang H., Yan B., Alberich-Jorda M., Zhang J., de Figueiredo-Pontes L.L., Battelli C. (2016). Targeted BMI1 inhibition impairs tumor growth in lung adenocarcinomas with low CEBPalpha expression. Sci. Transl. Med..

[73] Peer E., Tesanovic S., Aberger F. (2019). Next-Generation Hedgehog/GLI Pathway Inhibitors for Cancer Therapy. Cancers.

[74] Ain K., Taylor K., Rofiq S., Venkataraman G. (1997). Somatostatin Receptor Subtype Expression in Human Thyroid and Thyroid Carcinoma Cell Lines. J. Clin. Endocrinol Metab..

