# Supplementary material for: Targeting the Sonic Hedgehog Pathway to Suppress the Expression of the Cancer Stem Cell (CSC)—Related Transcription Factors and CSC-Driven Thyroid Tumor Growth
Source: Cancers (Basel). 2021 Jan 22;13(3):418. doi: 10.3390/cancers13030418 (PMC7866109; doi:10.3390/cancers13030418)
Supplement: Supplementary file 1 [file cancers-13-00418-s001.zip › cancers-997204-suppl-final/cancers-997204-suppl.pdf]

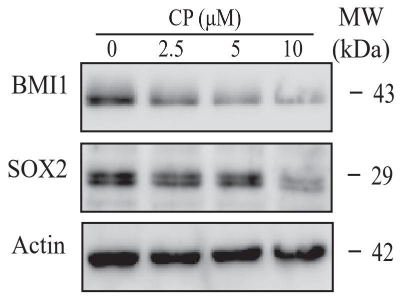

Fig. S1. Cyclopamine inhibits BMI1 and SOX2 expression in WRO82 cells. WRO82 cells were incubated for 48 hr in the absence or presence of the indicated concentrations of cyclopamine (CP). Cell lysates were prepared and analyzed for BMI1, SOX2, and actin expression by Western blot.

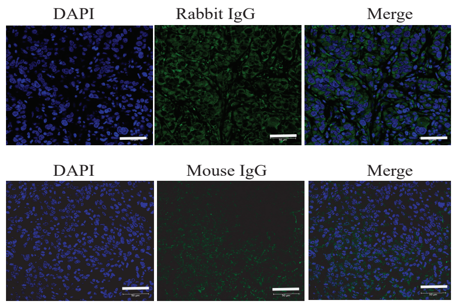

Fig. S2. Immunofluorescence staining with negative control antibodies. The frozen sections of tumor tissues from GANT61-treated mice were stained with normal rabbit or mouse IgG followed by incubating with Alexa Fluor-488-conjugated goat anti-rabbit and anti-mouse IgG, respectively. Scale bar, 50 μm.

A

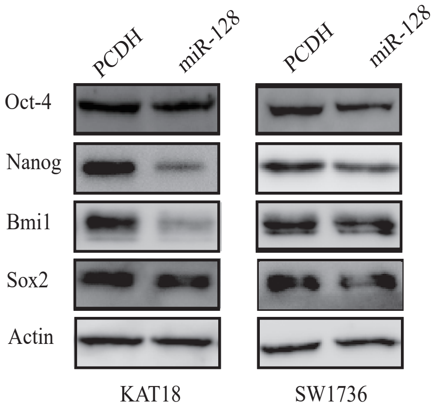

B

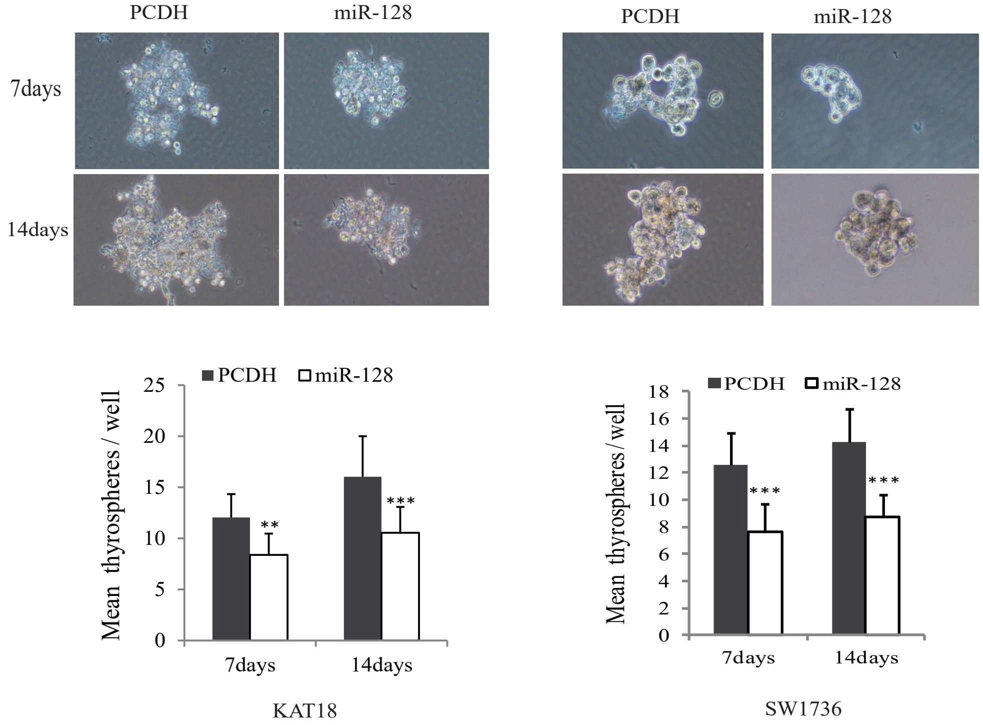

Fig. S3. miR-128 suppresses thyroid cancer stem cell self-renewal. (A) miR-128 down-regulates BMI1 expression. KAT-18 and SW1736 cells were stably transfected with the empty pCDH expression vector or the vector encoding miR-128. The cell lysates were analyzed for the levels of protein expression with the indicated antibodies. (B) miR-128 decreases thyrosphere formation. KAT-18 and SW1736 cells stably transfected with the empty pCDH expression vector or the vector encoding miR-128 were resuspended in serum-free DMEM/Ham's F-12 (1:1) medium containing B-27, EGF, and bFGF 20 ng/mL each and seeded at 2 x10^3 cells per well in six-well ultralow-attachment plates. A representative of thyrospheres from these transfected cell lines was photographed 2 weeks after seeding. Thyrospheres were counted and presented as the mean ± SD from one of two experiments in triplicate. ** $P < 0.01$; *** p<0.001.
