# Supplementary material for: Targeting the Sonic Hedgehog Pathway to Suppress the Expression of the Cancer Stem Cell (CSC)—Related Transcription Factors and CSC-Driven Thyroid Tumor Growth
Source: Cancers (Basel). 2021 Jan 22;13(3):418. doi: 10.3390/cancers13030418 (PMC7866109; doi:10.3390/cancers13030418)
Supplement: Supplementary file 1 [file cancers-13-00418-s001.zip › cancers-997204-suppl-final/File S1.zip!/File S1/Fig1-2.pdf]

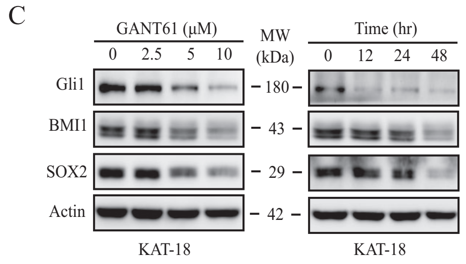
C
GANT61 (μM)
0 2.5 5 10
MW (kDa)
Time (hr)
0 12 24 48
Gli1
BMI1
SOX2
Actin
180
43
29
42
KAT-18
KAT-18

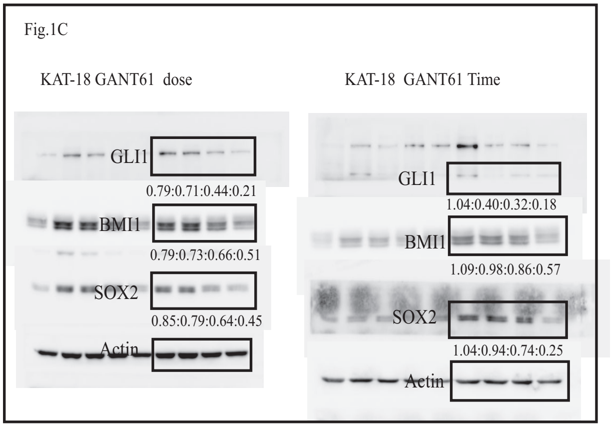
Fig.1C
KAT-18 GANT61 dose
KAT-18 GANT61 Time
GLI1
0.79:0.71:0.44:0.21
BMI1
0.79:0.73:0.66:0.51
SOX2
0.85:0.79:0.64:0.45
Actin
GLI1
1.04:0.40:0.32:0.18
BMI1
1.09:0.98:0.86:0.57
SOX2
1.04:0.94:0.74:0.25
Actin

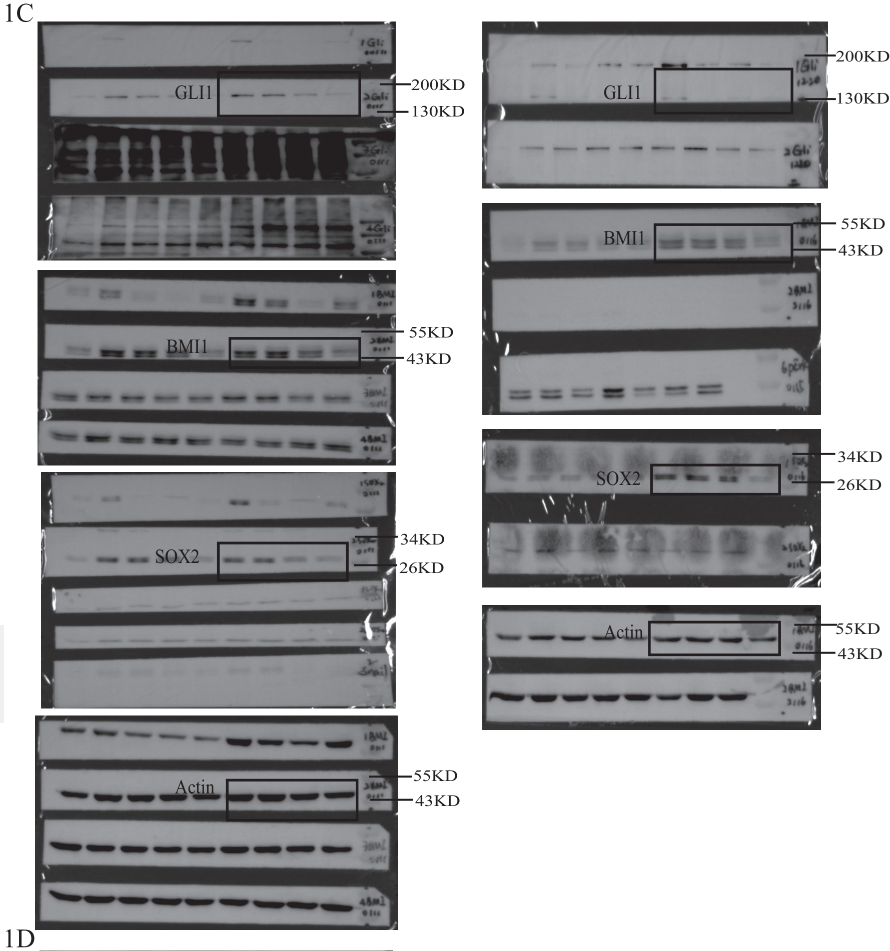
1C
GLI1
200KD
130KD
BMI1
55KD
43KD
SOX2
34KD
26KD
Actin
55KD
43KD
GLI1
200KD
130KD
BMI1
55KD
43KD
SOX2
34KD
26KD
Actin
55KD
43KD

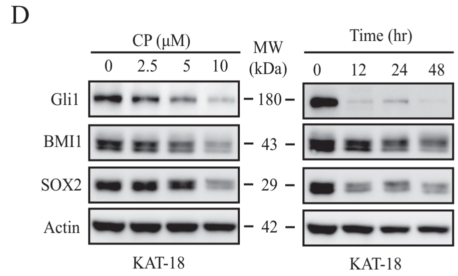
D
CP (μM)
0 2.5 5 10
MW (kDa)
Time (hr)
0 12 24 48
Gli1
BMI1
SOX2
Actin
180
43
29
42
KAT-18
KAT-18

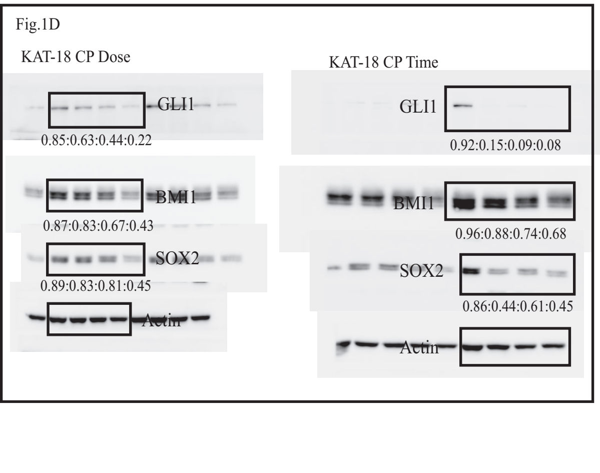
Fig.1D
KAT-18 CP Dose
KAT-18 CP Time
GLI1
0.85:0.63:0.44:0.22
BMI1
0.87:0.83:0.67:0.43
SOX2
0.89:0.83:0.81:0.45
Actin
GLI1
0.92:0.15:0.09:0.08
BMI1
0.96:0.88:0.74:0.68
SOX2
0.86:0.44:0.61:0.45
Actin

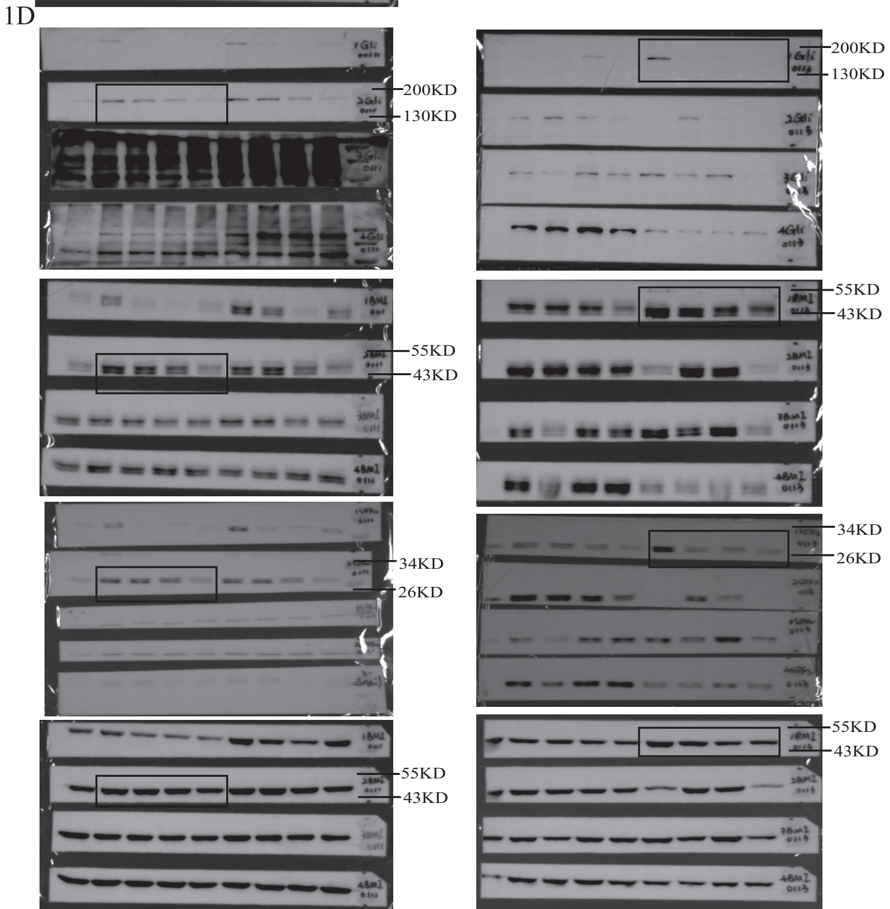
1D
200KD
130KD
55KD
43KD
34KD
26KD
55KD
43KD
200KD
130KD
55KD
43KD
34KD
26KD
55KD
43KD
