# Supplementary material for: Targeting the Sonic Hedgehog Pathway to Suppress the Expression of the Cancer Stem Cell (CSC)—Related Transcription Factors and CSC-Driven Thyroid Tumor Growth
Source: Cancers (Basel). 2021 Jan 22;13(3):418. doi: 10.3390/cancers13030418 (PMC7866109; doi:10.3390/cancers13030418)
Supplement: Supplementary file 1 [file cancers-13-00418-s001.zip › cancers-997204-suppl-final/File S1.zip!/File S1/Fig1.pdf]

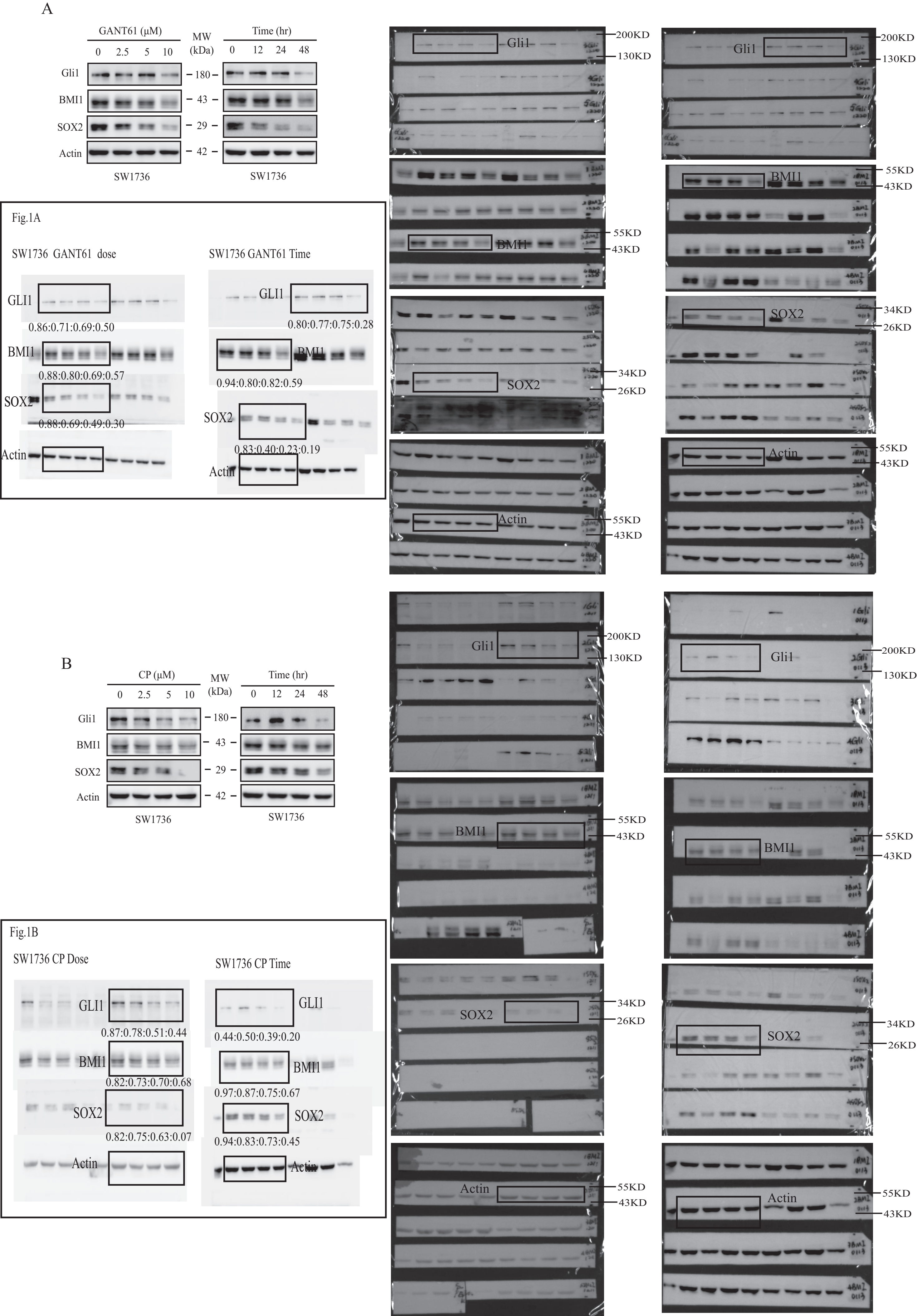
A
GANT61 (μM)
0 2.5 5 10
MW (kDa)
Time (hr)
0 12 24 48
Gli1
BMI1
SOX2
Actin
180
43
29
42
SW1736
SW1736
Fig.1A
SW1736 GANT61 dose
SW1736 GANT61 Time
GLI1
0.86:0.71:0.69:0.50
0.80:0.77:0.75:0.28
BMI1
0.88:0.80:0.69:0.57
0.94:0.80:0.82:0.59
SOX2
0.88:0.69:0.49:0.30
0.83:0.40:0.23:0.19
Actin
200KD
130KD
55KD
43KD
34KD
26KD
B
CP (μM)
0 2.5 5 10
MW (kDa)
Time (hr)
0 12 24 48
Gli1
BMI1
SOX2
Actin
180
43
29
42
SW1736
SW1736
Fig.1B
SW1736 CP Dose
SW1736 CP Time
GLI1
0.87:0.78:0.51:0.44
0.44:0.50:0.39:0.20
BMI1
0.82:0.73:0.70:0.68
0.97:0.87:0.75:0.67
SOX2
0.82:0.75:0.63:0.07
0.94:0.83:0.73:0.45
Actin
