# Supplementary material for: Targeting the Sonic Hedgehog Pathway to Suppress the Expression of the Cancer Stem Cell (CSC)—Related Transcription Factors and CSC-Driven Thyroid Tumor Growth
Source: Cancers (Basel). 2021 Jan 22;13(3):418. doi: 10.3390/cancers13030418 (PMC7866109; doi:10.3390/cancers13030418)
Supplement: Supplementary file 1 [file cancers-13-00418-s001.zip › cancers-997204-suppl-final/File S1.zip!/File S1/Fig2-1 -WB-update.pdf]

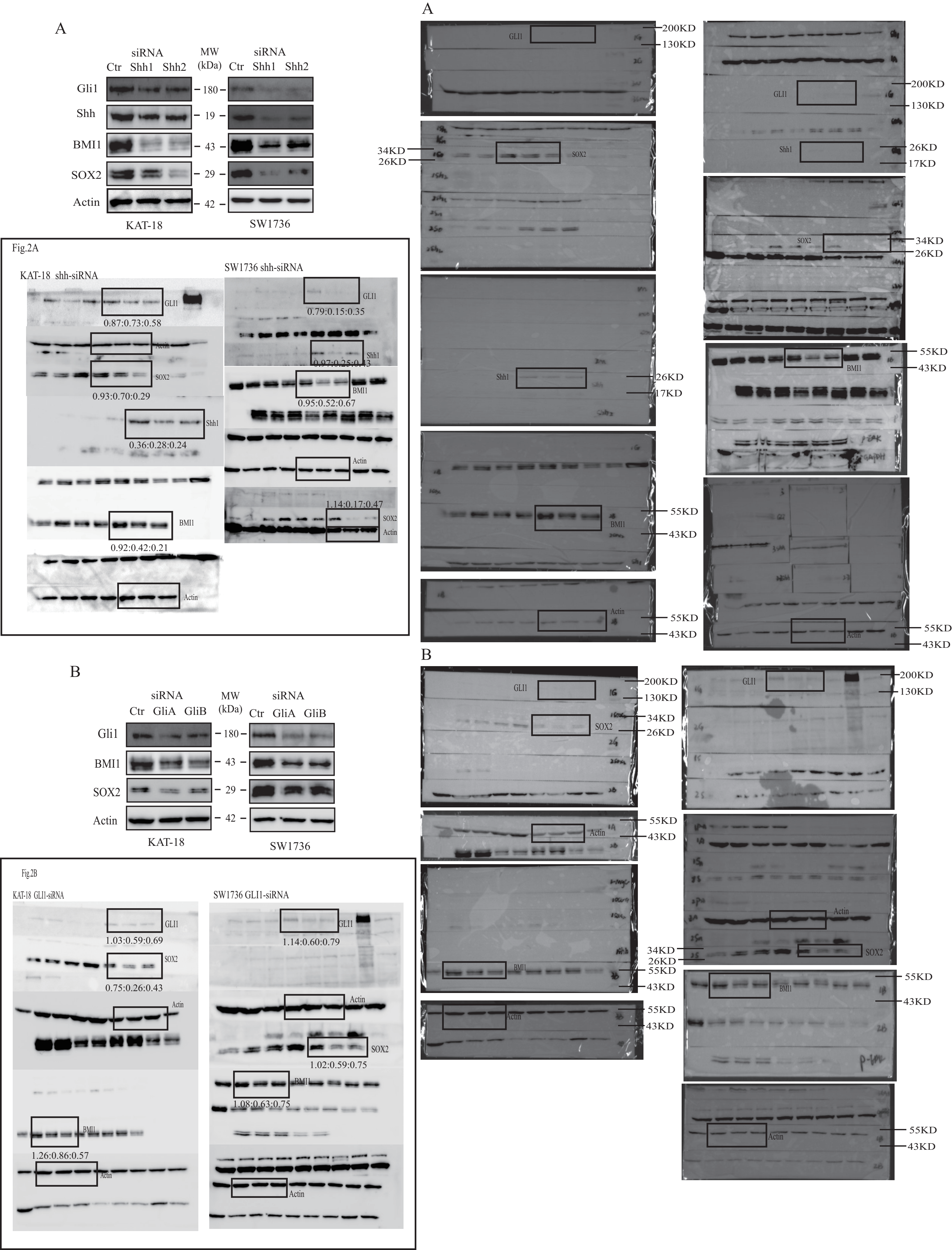
A
siRNA
Ctr Shh1 Shh2
MW (kDa)
siRNA
Ctr Shh1 Shh2
Gli1
Shh
BMI1
SOX2
Actin
180
19
43
29
42
KAT-18
SW1736
Fig.2A
KAT-18 shh-siRNA
SW1736 shh-siRNA
GLI1
0.87:0.73:0.58
Actin
SOX2
0.93:0.70:0.29
Shh1
0.36:0.28:0.24
BMI1
0.92:0.42:0.21
Actin
0.79:0.15:0.35
0.97:0.25:0.43
BMI1
0.95:0.52:0.67
1.14:0.17:0.47
B
siRNA
Ctr GliA GliB
Gli1
BMI1
SOX2
Actin
Fig.2B
KAT-18 GLI1-siRNA
SW1736 GLI1-siRNA
1.03:0.59:0.69
0.75:0.26:0.43
1.26:0.86:0.57
1.14:0.60:0.79
1.02:0.59:0.75
1.08:0.63:0.75
200KD
130KD
34KD
26KD
17KD
55KD
43KD
