# Supplementary material for: Targeting the Sonic Hedgehog Pathway to Suppress the Expression of the Cancer Stem Cell (CSC)—Related Transcription Factors and CSC-Driven Thyroid Tumor Growth
Source: Cancers (Basel). 2021 Jan 22;13(3):418. doi: 10.3390/cancers13030418 (PMC7866109; doi:10.3390/cancers13030418)
Supplement: Supplementary file 1 [file cancers-13-00418-s001.zip › cancers-997204-suppl-final/File S1.zip!/File S1/Fig2-2-WB-update.pdf]

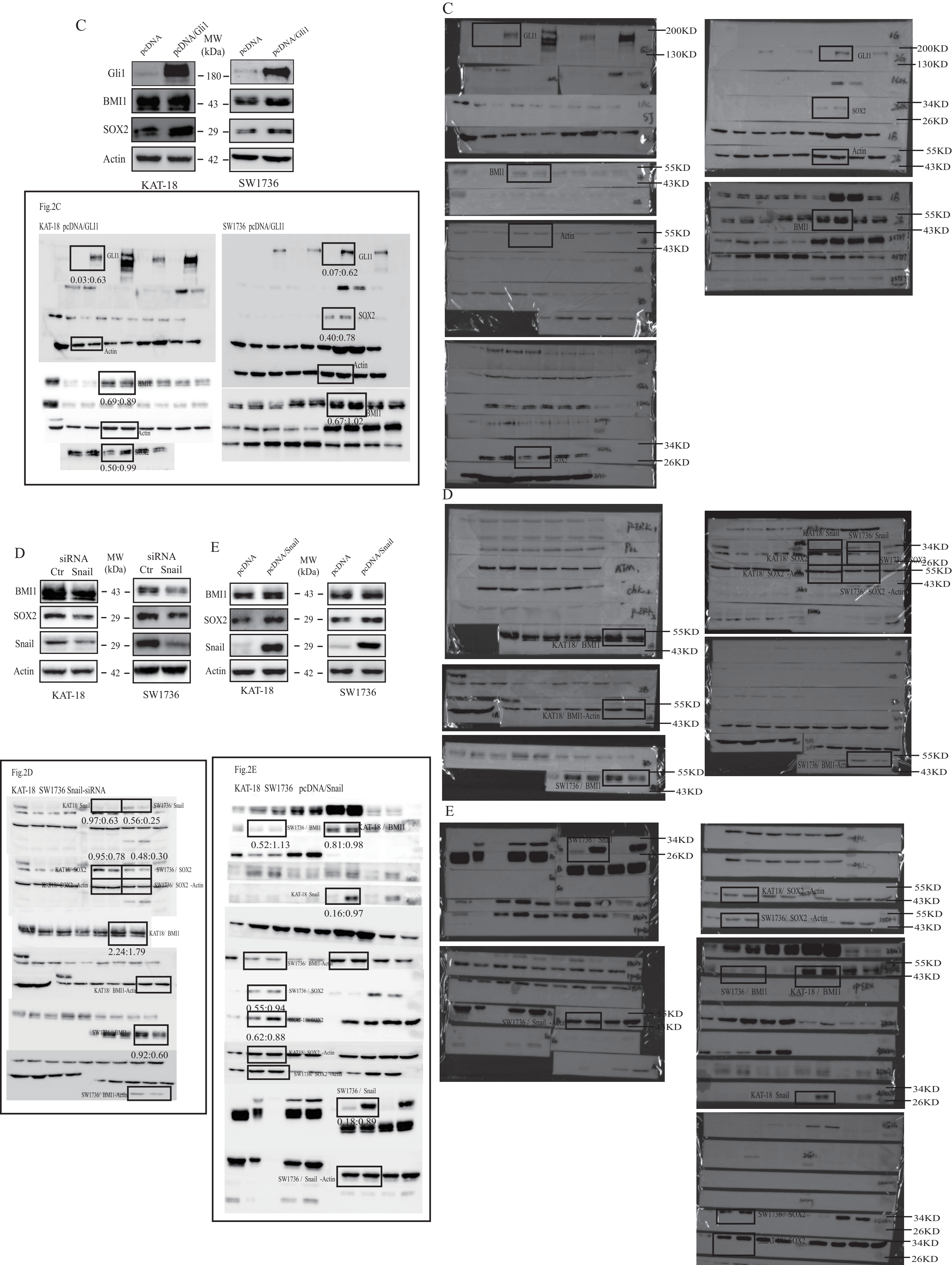
C
pcDNA
pcDNA/Gli1
MW (kDa)
Gli1
BMI1
SOX2
Actin
180
43
29
42
KAT-18
SW1736
Fig.2C
KAT-18 pcDNA/GLI1
SW1736 pcDNA/GLI1
0.03:0.63
0.07:0.62
0.40:0.78
0.69:0.89
0.67:1.02
0.50:0.99
D
siRNA
Ctr
Snail
E
pcDNA/Snail
Fig.2D
KAT-18 SW1736 Snail-siRNA
0.97:0.63
0.56:0.25
0.95:0.78
0.48:0.30
2.24:1.79
0.92:0.60
Fig.2E
KAT-18 SW1736 pcDNA/Snail
0.52:1.13
0.81:0.98
0.16:0.97
0.55:0.94
0.62:0.88
0.18:0.89
200KD
130KD
55KD
43KD
34KD
26KD
KAT18/ BMI1
KAT18/ BMI1-Actin
SW1736 / BMI1
SW1736/ BMI1-Actin
KAT18/ Snail
SW1736/ Snail
KAT18/ SOX2
KAT18/ SOX2 -Actin
SW1736/ SOX2 -Actin
SW1736/ SOX2
SW1736 / Snail -Actin
KAT-18 / BMI1
KAT-18 Snail
