# Supplementary material for: Targeting the Sonic Hedgehog Pathway to Suppress the Expression of the Cancer Stem Cell (CSC)—Related Transcription Factors and CSC-Driven Thyroid Tumor Growth
Source: Cancers (Basel). 2021 Jan 22;13(3):418. doi: 10.3390/cancers13030418 (PMC7866109; doi:10.3390/cancers13030418)
Supplement: Supplementary file 1 [file cancers-13-00418-s001.zip › cancers-997204-suppl-final/File S1.zip!/File S1/Fig5-WB-update eps.pdf]

Fig5

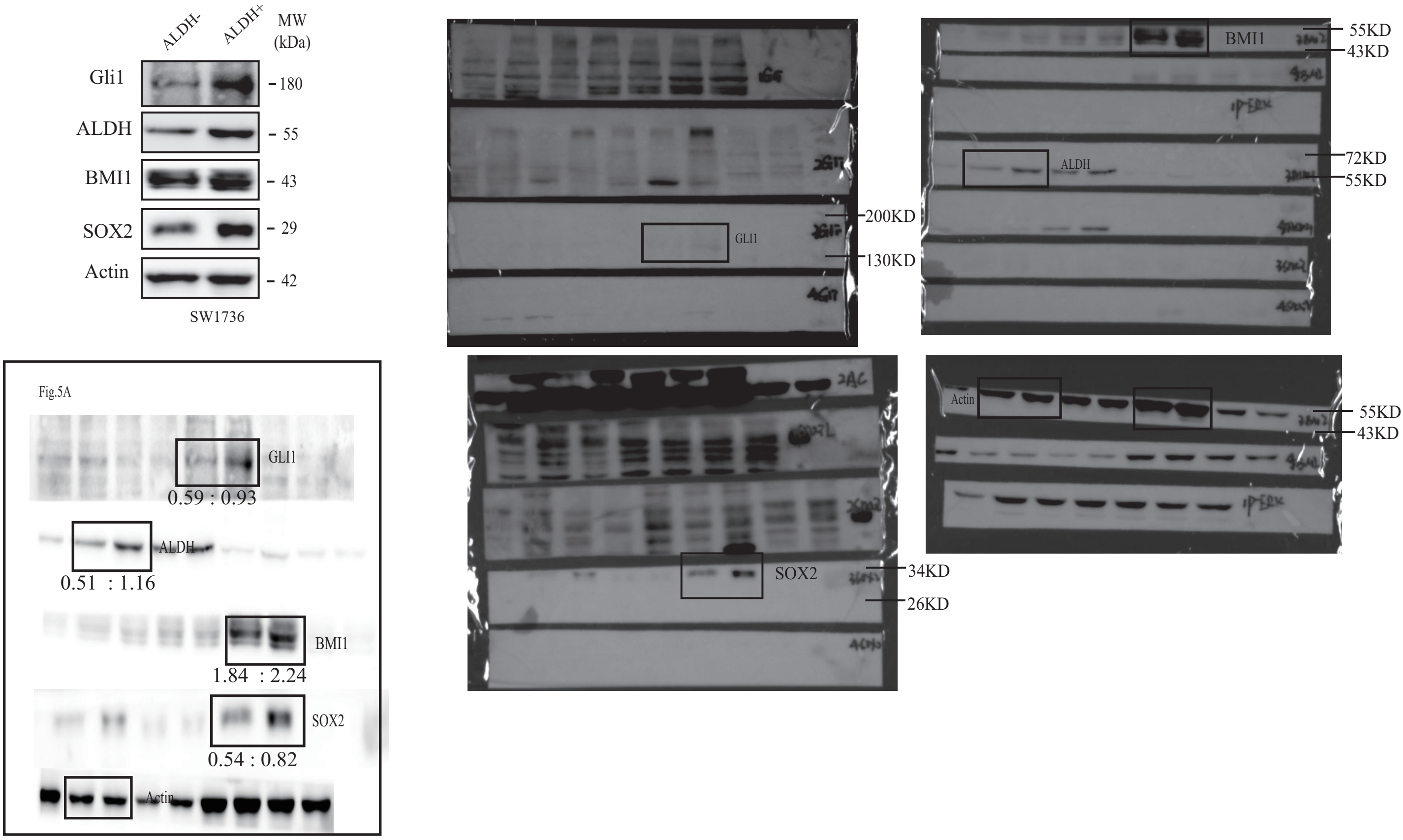
ALDH-
ALDH+
MW (kDa)
Gli1
- 180
ALDH
- 55
BMI1
- 43
SOX2
- 29
Actin
- 42
SW1736
Fig.5A
GLI1
0.59 : 0.93
ALDH
0.51 : 1.16
BMI1
1.84 : 2.24
SOX2
0.54 : 0.82
Actin
GLI1
200KD
130KD
BMI1
55KD
43KD
ALDH
72KD
55KD
SOX2
34KD
26KD
Actin
55KD
43KD

D

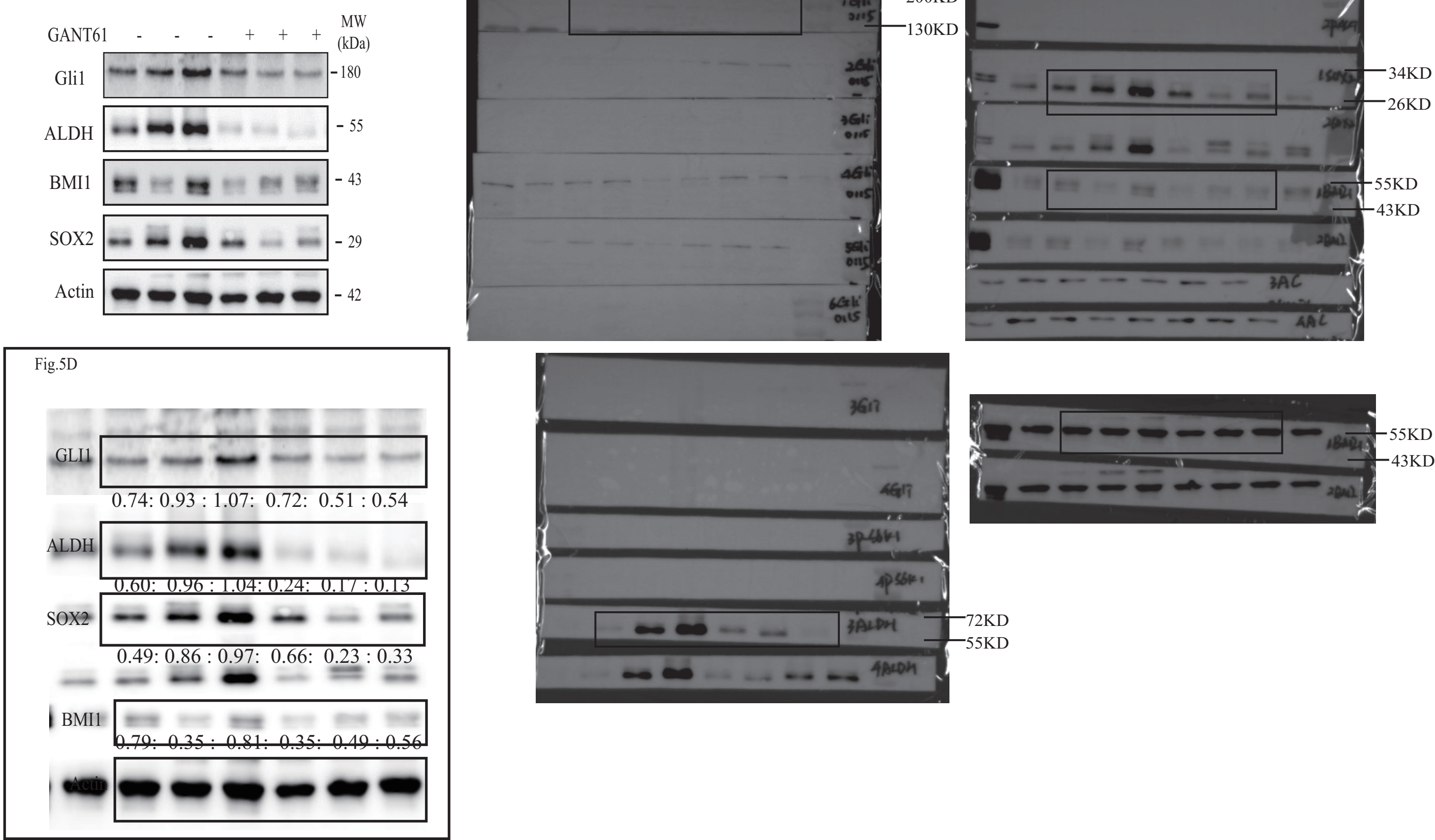
GANT61 - - - + + +
MW (kDa)
Gli1
- 180
ALDH
- 55
BMI1
- 43
SOX2
- 29
Actin
- 42
Fig.5D
GLI1
0.74: 0.93 : 1.07: 0.72: 0.51 : 0.54
ALDH
0.60: 0.96 : 1.04: 0.24: 0.17 : 0.13
SOX2
0.49: 0.86 : 0.97: 0.66: 0.23 : 0.33
BMI1
0.79: 0.35 : 0.81: 0.35: 0.49 : 0.56
Actin
200KD
130KD
34KD
26KD
55KD
43KD
72KD
55KD
55KD
43KD
